# Characterization of myocardial infarction by *in vivo* chemical exchange saturation transfer magnetic resonance imaging using natural D-glucose

**DOI:** 10.1016/j.jocmr.2025.102667

**Published:** 2025-11-30

**Authors:** Ajay Peddi, Daniel Schache, Chris Lippe, Sai Kiran Reddy Samawar, Michael Kuhlmann, Peter Niehaus, Jens Soltwisch, Emily Hoffmann, Ali Nahardani, Stephan Niland, Noelia Alonso Gonzalez, Klaus Dreisewerd, Uwe Karst, Lydia Sorokin, Michael Schaefers, Moritz Wildgruber, Cornelius Faber, Verena Hoerr

**Affiliations:** a Translational Research Imaging Center, Clinic of Radiology, University of Muenster, Muenster, Germany; b Institute of Physiological Chemistry and Pathobiochemistry, University of Muenster, Muenster, Germany; c European Institute for Molecular Imaging, University of Muenster, Muenster, Germany; d Institute of Inorganic and Analytical Chemistry, University of Muenster, Muenster, Germany; e Institute for Hygiene, Biomedical mass spectrometry, University of Muenster, Muenster, Germany; f Heart Center Bonn, Department of Internal Medicine II, University Hospital Bonn, Bonn, Germany; g Institute of Immunology, University of Muenster, Muenster, Germany; h Department for Radiology, University Hospital, LMU Munich, Munich, Germany

**Keywords:** Myocardial infarction, Remote myocardium, MRI, Gadolinium-based contrast agents, CEST, Late gadolinium enhancement

## Abstract

**Background:**

Cardiovascular magnetic resonance (CMR), the gold standard approach for characterizing myocardial infarction (MI), frequently relies on late gadolinium enhancement (LGE) using gadolinium-based contrast agents (GBCA). Whereas novel GBCAs targeting specific molecules have not yet entered clinical practice, chemical exchange saturation transfer (CEST) MRI shows promise for detecting various endogenous molecules. This study explored the potential of natural D-glucose as a biodegradable MRI contrast agent for imaging MI on day 7 by employing glucose-weighted CEST MRI (glucoCEST).

**Methods and results:**

In vivo, the application of cardiac glucoCEST MTR_asym_ (magnetization transfer ratio asymmetry) mapping delineated distinct pre- and postglucose infusion states in both healthy (n = 8) and MI-induced mice (n = 6) at 9.4T. This approach resulted in significant alterations in glucoCEST contrast, effectively identifying MI regions analogous to conventional LGE and immunohistochemical staining. *Ex vivo* mass spectrometry imaging confirmed elevated ^13^C-glucose and gadolinium accumulation in the MI region after exogenous administration, suggesting the potential of glucoCEST MRI for MI detection.

**Conclusion:**

Our preclinical study on MI demonstrated that cardiac glucoCEST MRI utilizing natural D-glucose as a biodegradable contrast agent effectively differentiates between MI regions, remote myocardium (RM), and healthy myocardium. The results were comparable to those obtained using LGE imaging.

## Graphical abstract

**Figure:**
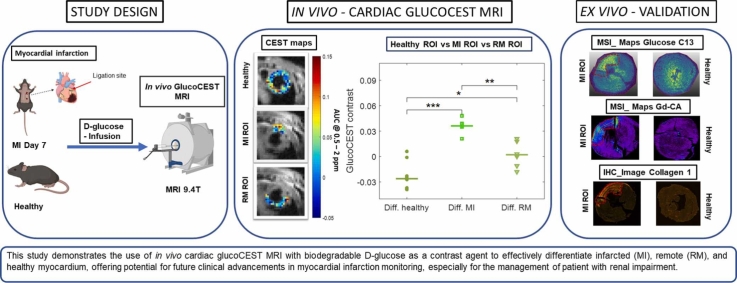

## 1 Introduction

In clinical settings, cardiovascular magnetic resonance (CMR) shows strong diagnostic value and is usually performed as the gold standard for myocardial tissue characterization, especially in myocardial infarction (MI) [1]. In recent years, many studies have shown that magnetic resonance imaging (MRI) can non-invasively assess the area at risk, infarct size, and myocardial salvage [2], [3]. It effectively captures the diverse stages of MI—acute, subacute, and chronic—by native T_1_ and T_2_ weighted imaging and mapping [4]. Elevated T_2_ relaxation times can be directly correlated with increased myocardial water content, reflecting edema and inflammation [1]. This quantitative marker is considered to be the first indication of myocardial injury, leading to decreased functionality and irreversible remodeling [5]. In contrast, late gadolinium enhancement (LGE) [6] MRI acquired through T_1_ weighted imaging and often regarded as the gold standard for assessing myocardial viability and detecting fibrosis, allows for the determination of the transmural extent of infarction by enabling precise visualization of scar tissue distribution [2], [7].

In addition to the commonly used non-targeted gadolinium-based contrast agents (GBCAs), advancements have emerged in agents targeting specific molecular markers in MI, including serum albumin and extracellular matrix proteins such as elastin, tropoelastin, collagen, fibrin, and fibronectin, as well as epidermal growth factor receptor and protein tyrosine phosphatase [8]. Imaging these targets has deepened our understanding of their biological roles in basic MI research and has offered potential improvements in diagnostic accuracy [9] and therapeutic decision-making [10]. Unfortunately, most of the newly developed targeted gadolinium based contrast agents (GBCA) are still in clinical trials, mainly in phases I and II [8], [9] and given the rigorous demands inherent in translating *in vivo* applications into clinical practice, the relatively low success rate is not surprising [8]. Although GBCAs are generally effective, their potential toxicity may lead to adverse reactions, particularly in patients with renal impairment or hypersensitivities [11], [12].

To overcome these limitations, researchers have developed non-invasive molecular MRI techniques based on chemical exchange saturation transfer (CEST) to image endogenous metabolites and molecules, such as creatine (CrEST), in MI over the last decades [13], [14]. In CEST experiments, labile protons with a chemical shift distinct from water are selectively saturated and transfer saturation to the bulk water [15]. Thus, CEST is sensitive to small metabolites and macromolecules containing moieties with labile protons, such as those found in amine, amide, or hydroxyl functional groups [16]. While CrEST imaging is a non-specific imaging approach, Vandsburger et al. developed an *in vivo* cardiac CEST MRI contrast specifically for imaging the chronic phase of MI based on endogenous fibrosis, targeting a resonance frequency at −1 ppm from the water resonance frequency [17].

In 2012 and 2013, Chan et al. and Walker-Samuel et al. [18], [19] demonstrated in a tumor mouse model that D-glucose is a suitable biodegradable CEST MRI contrast agent based on five exchangeable hydroxyl groups. To date, D-glucose has been detected inside tissues *in vivo* by relaxometry (T_1rho_ and T_2_) [20], [21], [22] or chemical exchange-related approaches such as CEST and CESL (Chemical exchange-sensitive spin-lock) [22], [23], [24]. Glucose-weighted CEST (glucoCEST) contrast post-administration of D-glucose or glucose analogs has been effectively monitored in various animal models, including tumors [25], kidney rejection [26], intrauterine inflammation [27], and brain injuries [28]. Promising results have also been demonstrated in clinical studies investigating cerebral ischemia, neurological disorders, lymphedema, osteoarthritis, muscle physiology, and solid tumors [29], [30], [31]. However, applying CEST MRI to a dynamic heart, especially a fast-beating mouse heart, presents challenges, necessitating specialized techniques. Researchers such as Zhou et al. [32], Vandsburger et al. [17], and Schache et al. [33] successfully introduced distinct cardiac CEST MRI methods, laying the groundwork for glucoCEST applications in myocardial imaging.

This study aimed to comprehensively explore the feasibility and potential of *in vivo* CEST MRI using natural D-glucose as a contrast agent to assess the remodeling processes with respect to infarction size, metabolic and morphological changes, and border zones in the infarcted myocardium. Our findings were confirmed by *in vivo* LGE MRI and *ex vivo* immunohistochemistry (IHC) staining methods, as well as by mass spectrometry imaging to visualize ^13^C-glucose and quantify Gd distribution in the MI region.

## 2 Methods

### 2.1 Study design

**Fig. 1:**
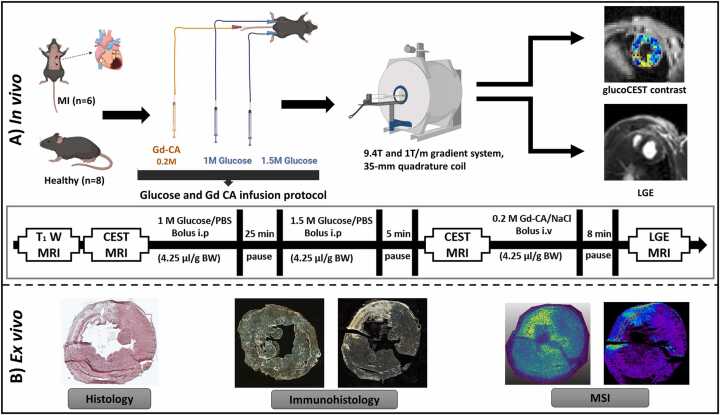
Experimental outline. **(A)***In vivo* examinations: Female C57BL/6 J mice, both healthy and those induced with MI (Day 7), were investigated by i*n vivo* cardiac MRI. glucoCEST MRI in comparison to LGE was conducted by using natural D-glucose and the Gd-CA gadofosveset, respectively. **(B)***Ex vivo* experiments: After MRI, the mice were euthanized for comprehensive heart tissue analysis, which involved identifying infarct regions through standard histology and immunohistochemistry techniques. Furthermore, we conducted quantitative analysis of local Gd and ^13^C-labeled/^12^C-unlabeled glucose concentrations using cutting-edge LA-ICP-MSI and quantitative MALDI-2-MSI methods, respectively. *MI* myocardial infarction, *MRI* magnetic resonance imaging, *glucoCEST* glucose-weighted chemical exchange saturation transfer, *LGE* late gadolinium enhancement, *Gd-CA* gadolinium contrast agent, *MALDI-2-MSI* matrix-assisted laser desorption/ionization mass spectrometry imaging with laser post-ionization, *LA-ICP-MSI* laser ablation inductively coupled plasma mass spectrometry imaging

This imaging study on MI comprised two experimental phases: *in vivo* and *ex vivo* (Fig. 1). In the *in vivo* phase, animals with MI (n = 6) were investigated in comparison to healthy controls (n = 8) using *in vivo* MRI on day 7 post-surgery. CEST and LGE MRI were performed on a 9.4T small animal MRI scanner using D-glucose and gadofosveset as contrast agents, each with dedicated time-point infusion protocols. In the *ex vivo* phase, CEST MRI data were validated using histological examination, IHC, laser ablation inductively coupled plasma mass spectrometry (LA-ICP-MS), and matrix-assisted laser desorption/ionization mass spectrometry imaging with laser post-ionization (MALDI-2-MSI). By using ^13^C_6_-labeled glucose (u-^13^C-glucose), it was possible to distinguish between glucose administered as a contrast agent and intrinsic glucose in the corresponding MSI maps.

### 2.2 Animal experiments and mouse model

All experimental and animal husbandry procedures were conducted strictly in accordance with the German Animal Welfare Act and local animal welfare guidelines and had received full approval from the Landesamt für Natur, Umwelt und Verbraucherschutz NRW (Protocol No. 81–02.04.2019. A169). Our study utilized eight-week-old female C57BL/6 mice procured from Charles River Laboratories (Sulzberg, Germany). This age group was selected to ensure developmental maturity and was aligned with the specific objectives of our research. Throughout the study period, mice were housed in a controlled environment designed to mimic natural lighting conditions, following a 12-hour light-dark cycle. The study included two groups of mice weighing 20–25 g: healthy animals (n = 8) and animals with MI (n = 6). During surgery, anesthesia was induced with 2% isoflurane and a mix of O_2_/medical air at a flow rate of 1 L/min. The animals underwent tracheal intubation for ventilation using a dedicated small-animal mini-vent ventilator model-845 (Hugo Sacks Electronic, Germany). MI was induced in mice through a left thoracotomy and permanent ligation of the left coronary artery using an 8–0 Ethilon suture. Successful ligation was confirmed by regional blanching of the left ventricle and the apex [34]. After surgery, all MI animals were closely monitored and underwent MRI scanning 7 days post-surgery to assess the extent of MI. Animals received a combination of analgesia and anesthesia (0.04 mg Fentanyl/kg and 4 mg Midazolam/kg) by intraperitoneal (i.p.) administration preoperatively. During the recovery period, the analgesic carprofen (0.005 mg carprofen/g) was injected subcutaneously on three successive days. After MRI scans, the animals were euthanized by cervical dislocation for *ex vivo* analysis.

### 2.3 *In vivo* MRI

*In vivo* MRI measurements were conducted using a 9.4T small animal MRI system (BioSpec 94/20, Bruker Biospin, Ettlingen, Germany) equipped with a 1 T/m gradient system and controlled by ParaVision 6.0.1 software. A 35 mm volume resonator coil was used for signal transmission and reception. Throughout the experiments, MRI procedures were carried out under isoflurane anesthesia (1.5%–2.5% isoflurane and 0.7/0.3 medical air/O_2_ mixture), with continuous monitoring of core body temperature, electrocardiogram, and respiration rates.

All applied sequences and acquisition parameters are summarized in Table 1. CEST contrast measurements were conducted in the short-axis view using a modified cardiac (ECG) and respiratory-triggered FLASH (fast low-angle shot) sequence with an implemented CEST module. T_2_ relaxation time maps were obtained using a multi-echo RARE (rapid acquisition with relaxation enhancement) experiment, whereas for LGE MRI, T_1_-weighted images were acquired using an Ig-FLASH (Intragate-FLASH) sequence with variable flip angles of 2°, 5°, 8°, 11°, and 14°.

**Table 1:** Overview of MRI parameters used for different sequences in cardiac *in vivo* experiments.

Parameters	GlucoCEST mapping (FLASH)	T_1_ Mapping/T1w (Ig FLASH)	T_2_ Mapping (RARE)
Flip angle	15^o^	14^o^ 11^o^ 8^o^ 5^o^ 2^o^ /15^o^	-
TE/TR	2.3/398 ms	1.7/5.8 ms	11.30, 33.89, 56.48, 79.07, 101.66, 124.26 /1000 ms
FOV	30 × 30 mm^2^	30 × 30 mm^2^	30 × 30 mm^2^
Slice thickness	1 mm	1 mm	1 mm
Matrix	96 ×96	128 ×128	96 ×96
Averages	2	1	4
Magnetization transfer module	Single block pulse, T_sat_ = 140 ms, B1 = 2 μT	-	-
Saturation offset range	±4 ppm	-	-
Saturation steps	200 Hz (21 steps in total); Interleaved from higher offsets to zero [Hz]: −2000, +2000, −1800, +1800, −1600, +1600, −1400, +1400, −1200, +1200, −1000, +1000, −800, +800, −600, +600, −400, +400, −200, +200, 0	-	-
S_O_ offset	±15 ppm	-	-
Approximate scan time	20 min	3 min	48 s

*MRI* magnetic resonance imaging, *glucoCEST* glucose-weighted chemical exchange saturation transfer, *TE* echo time, *TR* repetition time, *FOV* field of view

### 2.4 In vitro glucose experiments

To validate our results and the MRI glucoCEST sequence glucose was dissolved on PBS in different concentrations (1 mM to 75 mM). The pH was maintained at 7.4. The same glucoCEST MRI protocol was used as for *in vivo* experiments. The phantoms were measured at room temperature at 18 °C to 20 °C, controlled by a temperature probe.

### 2.5 Infusion protocols for contrast agents

For glucoCEST MRI, a glucose infusion protocol was established by optimizing an appropriate glucose infusion scheme. A detailed description of the validation of the glucose infusion protocol is provided in Supplemental Fig. 1. Before the measurements, the animals received a bolus i.p injection of a 1.0 M glucose/PBS solution (4.25 µL/g body weight) followed by a second bolus i.p. injection of a 1.5 M glucose/PBS solution (4.25 µL/g body weight) after 25 min. Five minutes after the second glucose administration, glucoCEST measurements were performed [26]. This protocol successfully raised blood glucose levels from about 200 mg/dl (before glucose infusion; without fasting) to approximately 400 mg/dl and maintained them consistently throughout the imaging session. As internal study control, for each animal, the blood glucose levels were measured by the glucometer Contour Next (Ascensia Diabetes Care Holding AG, Basel, Switzerland) before and directly after MRI.

For LGE MRI, a solution of 0.2 M gadofosveset trisodium (Ablavar; TMC Pharma Services Ltd, Hartley Wintney, Hampshire, UK) was administered intravenous (i.v) (4.25 µL/g body weight) 8 min before the acquisition of T_1_-weighted images (Fig. 1). Pin ports and 29-gauge cannulas (Intech Laboratories Europe GmbH, Germany) were used for the infusion tubes.

### 2.6 CEST MRI data analysis

CEST MRI data were analyzed pixel-wise using a custom-written MATLAB script (MATLAB R2018a, MathWorks, Natick, Massachusetts), which is publicly available through the GitHub repository [https://github.com/DrD1212/CardiacCEST]. Therefore, either one region of interest (ROI) was drawn covering the entire myocardium (for healthy animals) or two ROIs covering the zones of the MI and remote myocardium (RM) (for MI-induced animals) in the respective slice. A Z-spectrum was generated for each pixel before and after glucose infusion within the ROI by normalizing the signal intensities measured at each saturated frequency offset to the mean of both the S0 values (±15 ppm). B0-correction was followed by spline interpolation (4001 points) shifting the minimum of the Z- spectrum to the water resonance frequency. Additionally, DOwnsampling by SEparation of Z-spectrum into two parts (DOSE) filtering was applied to correct for Rabi oscillations induced by the short saturation pulse that was applied within the CEST module[33]. Subsequently, the magnetization transfer ratio asymmetry (MTR_asym_) was calculated pixel-wise before and after glucose infusion, and the pixel-wise glucoCEST contrast was determined by integrating the MTR_asym_ spectra within a frequency range of 0.5 to 2.0 ppm. Pixel-wise glucoCEST values were then superimposed on the anatomical images of the corresponding slices to generate glucose distribution maps (Supplemental Fig. 2). Finally, pixel-wise Z-spectra were averaged to generate a global Z-spectrum for the complete ROI, and the glucoCEST contrast was calculated in analogy to the pixel-wise analysis based on MTR_asym_ both before and after the glucose infusion:

$${\mathrm{MTR}}_{\mathrm{asym}}=\frac{S\left(-∆\omega \right)}{S_{0}}-\frac{S(∆\omega )}{S_{0}}$$

$$\mathrm{glucoCEST contrast pre glucose infusion}=\int_{0.5\mathrm{ppm}}^{2\mathrm{ppm}}{\mathrm{MTR}}_{\mathrm{asym}\_\mathrm{pre}}$$

$$\mathrm{glucoCEST contrast post glucose infusion}=\int_{0.5\mathrm{ppm}}^{2\mathrm{ppm}}{\mathrm{MTR}}_{\mathrm{asym}\_\mathrm{post}}$$

For the final statistical analysis, the difference in the glucoCEST contrast pre- and postglucose infusion (Δ glucoCEST) was calculated for different ROIs (healthy, MI, and RM regions) according to the following equation

$$∆\mathrm{glucoCEST contrast}=\;\int_{0.5\mathrm{ppm}}^{2\mathrm{ppm}}{\mathrm{MTR}}_{\mathrm{asym}\_\mathrm{post}}-\int_{0.5\mathrm{ppm}}^{2\mathrm{ppm}}{\mathrm{MTR}}_{\mathrm{asym}\_\mathrm{pre}}$$

**Lorentzian Difference Analysis** [35]

The following single Lorentzian model is fitted to the Z-spectra at the previously B0-corrected frequency offsets Δω to account for direct water saturation represented by the amplitude (a) and width (b) as well as a constant MT background (c):

$$Z_{\mathrm{ref}}\left(\Delta \omega \right)=c-a\cdot \frac{b^{2}/4}{b^{2}/4+\Delta \omega^{2}}$$

For further analysis, the residual magnetization transfer ratio (MTR_res_) has been calculated:

$$\mathrm{MT}R_{\mathrm{res}}\left(\Delta \omega \right)=Z_{\mathrm{ref}}\left(\Delta \omega \right)-Z\left(\Delta \omega \right)$$

The corresponding glucoCEST contrast was calculated as

$$\mathrm{glucoCEST contrast pre glucose infusion}=\int_{0.5\mathrm{ppm}}^{2\mathrm{ppm}}{\mathrm{MTR}}_{\mathrm{res}\_\mathrm{pre}}$$

$$\mathrm{glucoCEST contrast post glucose infusion}=\int_{0.5\mathrm{ppm}}^{2\mathrm{ppm}}{\mathrm{MTR}}_{\mathrm{res}\_\mathrm{post}}$$

### 2.7 Validation of the cardiac glucoCEST sequence

The cardiac CEST method is based on a recently published study by Schache et al. [33], which employed a short saturation pulse length of 140 ms and a short TR of 386 ms. Although the saturation effect of such short pulses is typically very low at long repetition times, a cumulative effect and pronounced MTR_asym_ contrast were observed in a 75 mM glucose phantom at short TR (Supplemental Fig. 3). This resulted in a strong correlation between glucoCEST contrast and glucose concentration (R² > 0.99; Supplemental Fig. 4) at concentrations above 10 mM.

It is well established that the physiological blood glucose concentration ranges from 5 to 10 mM [36], a level that lies below the detection limit of glucoCEST MRI, even in a PBS phantom (Supplemental Fig. 4A). The corresponding myocardial tissue glucose concentration is even lower and reaches approximately 50% of the plasma glucose concentration [37]. However, in hyperglycaemic conditions, data suggest that the glucose concentration in the MI core region showing residual flow but impaired washout (Supplemental Fig. 10A, B). can approach values near the blood glucose level [38]. Thus, in our experiments, when applying the proposed glucose infusion protocol (100 µL of 1 M glucose solution, 25 min waiting time, and 100 µL of 1.5 M glucose solution; Fig. 1), tissue glucose concentrations were estimated based on blood glucose levels during the glucoCEST MRI measurements, reaching values between 22 mM and 35 mM (Supplemental Fig. 1D).

To simulate the resulting glucose-induced contrast, a cardiac tissue model was generated before and after glucose infusion using Bloch McConnell simulations [39]. (Schuenke et al., https://github.com/schuenke/BMCTool). The cardiac tissue model consisted of water (T₁ = 1.429 s, T₂ = 0.029 s); MT (proton fraction = 0.01, T₁ = 1.0 s, T₂ = 4.0 × 10⁻⁵ s, exchange rate = 30 Hz, chemical shift = –3.0 ppm, line shape = Lorentzian); hydroxyl (proton fraction before glucose infusion = 0.00045 (10 mM), after glucose infusion = 0.000991 (22 mM), after glucose infusion = 0.00158 (35 mM), T₁ = 1.0 s, T₂ = 0.1 s, exchange rate = 3500 Hz, chemical shift = 1.5 ppm); amine (proton fraction = 0.000090 (10 mM), T₁ = 1.0 s, T₂ = 0.1 s, exchange rate = 5000 Hz, chemical shift = 3 ppm); and amide (proton fraction = 0.000135 (15 mM), T₁ = 1.0 s, T₂ = 0.1 s, exchange rate = 50 Hz, chemical shift = 3.5 ppm) pools. The corresponding simulated MTR_asym_ spectra are shown in Supplemental Fig. 4B, revealing a ΔglucoCEST contrast between 22 mM and 10 mM 0.010244 and between 35 mM and 10 mM 0.020956. Thus, already at a glucose level of 22 mM, a measurable ΔglucoCEST contrast is obtained.

### 2.8 Relaxation time analysis

For each defined ROI (healthy myocardium, MI, and RM), T_1_ relaxation times were calculated by fitting the mean signal values acquired with variable flip angles (2°, 5°, 8°, 11°, and 14°) to the Ernst equation (Supplemental Fig. 6A–C). In contrast, T_2_ relaxation times were quantified pixel-wise, and maps were created using MATLAB. For each defined ROI, the mean T_2_ relaxation time was calculated over all pixels within the ROI.

### 2.9 Statistical analysis software

All unpaired statistical group analyses were conducted using the Mann-Whitney U-test, while for paired group comparison the Wilcoxon signed-rank test was applied. GraphPad Prism (GraphPad Software Inc., San Diego, California), MATLAB, Excel (Microsoft, Inc. Redmond, Washington), ImageJ (https://imagej.net/ij/), and Bio Render (Biorender, Inc., Toronto, Ontario, Canada) were used for data analysis and visualization.

### 2.10 *Ex vivo* investigation

After acquiring MRI data, the animals were euthanized, and their hearts were isolated and sectioned into two parts. Both the base and apex of the hearts were embedded in the M-1 embedding matrix (Epredia, Richard-Allan Scientific LLC, Breda, The Netherlands) and shock-frozen using liquid nitrogen. Heart segments were cut using a cryotome (Leica CM 1850, Wetzlar, Germany) at a thickness of 10 µm mounted to microscopy slides (Superfrost, Thermo Fisher, Dreieich, Germany) and stored at −80 °C.

### 2.11 Immunohistochemistry and confocal microscopy

Sections directly taken from −80 °C storage were allowed to equilibrate at room temperature for 10 min before being fixed in pre-cooled methanol at −20 °C for another 10 min. Following the removal of methanol, the section boundaries were delineated using an Immedge pen to create a hydrophobic barrier. After drying, the sections underwent a 5-minute wash in PBS before blocking with a solution consisting of 1% (w/v) BSA and 0.5% (v/v) Triton X-100 in PBS for 45 min at room temperature. Subsequently, the primary antibodies (Table 2A) were diluted in the blocking solution and applied to the sections, which were incubated for 1 h at room temperature in a humid chamber. Next, the sections were rinsed three times with PBS for 10 min each, and secondary antibodies (Table 2B) were applied to the sections and incubated for 45 min at room temperature in a humid chamber. Excess and non-specifically bound antibodies were removed with a single 10-minute wash in PBS. Nuclear staining was then performed using DAPI (4',6-diamidino-2-phenylindole) for 10 min, followed by two additional 10-minute washing steps in PBS. Finally, the sections were mounted in elvanol, covered with a glass coverslip, and stored at 4 °C. Tile scan images and the corresponding high-magnification images were captured using a laser scanning confocal microscope (LSM 900, Zeiss, Jena, Germany). Image processing and exporting were carried out using ZEN 3.1 software (Zeiss).

**Table 2:** List of immunohistochemistry antibodies

A)				
Antigen	Description	Concentration	Dilution	Source/Cat. No.
CD206	Goat anti-mouse MMR/CD206	0.2 mg/mL	1:400	R&D Systems; AF2535
F4/80	eFluor 570 rat anti-mouse F4/80	0.2 mg/mL	1:500	Affymetrix/eBioscience; 41-4801
Pan Laminin	Rabbit anti-mouse laminin 111 (PAN-LN)	Serum	1:1000	Prepared in-house L.M.Sorokin
CD31	Hamster anti-mouse CD31 (PECAM-1), clone: 2H8	0.5 mg/mL	1:500	ThermoFischer; MA3105
PDGFRα	Goat anti-mouse PDGF receptor alpha,	0.2 mg/mL	1:500	R&D Systems; AF1062-SP
Isolectin GS-B4	Alexa Fluor 647 isolectin GS-B4 from Griffonia simplicifolia	1 mg/mL	1:200	Life Technologies; 132450
CD45	Rat anti-mouse CD45.2	Conditioned medium		Ralph and Berridge (1984); Prepared in-house L. M. Sorokin
Collagen I	Rabbit anti-mouse collagen I	1 mg/mL	1:200	Millipore/Chemicon; AB765P
Hexokinase 1	Rabbit anti-human Hexokinase 1	0.207 mg/mL	1:50	Abcam; ab150423
Connexin 37	Rabbit anti-mouse connexin 37	0.25 mg/mL	1:100	Thermo Fischer; 40-4300
Connexin 43	Rabbit anti-human connexin 43	0.25 mg/mL	1:100	Cell Signaling Technology; 3512

B)				
Antibody	Concentration	Fluorophore	Dilution	Source/Cat. No.
Donkey anti-goat IgG (H+L)	2 mg/mL	Alexa Fluor 488	1:400	ThermoFischer; A11055
Alexa Fluor 647 goat anti-hamster IgG (H+L)	2 mg/mL	Alexa Fluor 647	1:1000	Abcam; ab173004
Goat anti-rabbit IgG (H+l)	2 mg/mL	Alexa Fluor 488	1:600	Invitrogen; A11008
Donkey anti-rabbit IgG (H+L)	2 mg/mL	Alexa Fluor 594	1:1000	Invitrogen; A21207
Donkey anti-rabbit IgG (H+L)	2 mg/mL	Alexa Fluor 647	1:1000	Abcam; Ab150067
Donkey anti-rat IgG (H+L), pre-absorbed	2 mg/mL	Alexa Fluor 555	1:1000	Abcam; ab150155
Donkey anti-rat IgG (H+L), preadsorbed	2 mg/mL	Alexa Fluor 647	1:1000	Abcam; ab150155
Donkey anti-sheep IgG (H+L), cross-adsorbed	2 mg/mL	Alexa Fluor 488	1:1000	Invitrogen; A-11015

(A) Primary antibodies used for immunohistochemistry (IHC) of mouse heart. (B) Fluorophore-conjugated secondary antibodies were used for IHC fluorescence staining

### 2.12 Mass spectrometry imaging

#### 2.12.1 MALDI MSI

Tissue sections were dried in a desiccator, vacuum-sealed, and stored at −80 °C. At the time of measurement, the slides were brought to room temperature in an evacuated desiccator and coated with a Norharmane matrix for MALDI analysis using a pneumatic spray robot (Sun Collect MALDI Sprayer; Sun Chrome, Friedrichsdorf, Germany). The parameters were: 3,5 mg/ml norharmane solution in 50:50 (% V/V) acetonitrile: water; total of 20 passages, flow rate: first passage 15 µL/min; second passage 20 µL/min; third passage 30 µL/min; all following: 50 µL/min; scan speed: 600 mm/min, line distance 2 mm. MALDI-MS analysis was conducted on a TOF flex MALDI2 (Bruker Daltonics, Bremen, Germany) in the negative ion mode with microGRID. The parameters used were as follows: spot size, 14 × 29 µm²; step size (x and y), 30 µm; shots per pixel, 150; *m/z* range: 50–500; trigger delay for MALDI2, 10 µs. Similar to our previous work [40], a combination of tandem MS and MS images was used for the unambiguous identification of both types of glucose. Two MALDI-MS images were collected from the same ROI in each section with a pixel size of 30 µm. For each individual image, a pristine area of 14 × 29 µm² within a 30 × 30 µm² pixel was probed by shifting the ROI by 15 µm in the x-direction between the measurements. Both runs were collected in tandem MS mode by selecting the deprotonated ion species of glucose at *m/z* 179 (^12^C_6_-glucose) and 185 (^13^C_6_-glucose) as precursors. The precursor ions were fragmented using an activation energy of 8 eV for low-energy collision-induced dissociation (CID). Images were constructed and analyzed using the SCiLS Lab MVS (SCiLS, Bremen, Germany).

After the analysis, the matrix was washed with ethanol, and the samples were stained with H&E. ROIs for the analysis (healthy, MI, and RM) were identified based on the bright-field microscopy of the stained samples. The same ROI was used for both images acquired from the same section and transferred between SCiLS lab files. To compare the relative intensities of ^13^C-labeled and ^12^C-unlabeled glucose in both areas, a characteristic ring cleavage yielding fragment ions at 89.02 for ^12^C_6_-glucose and 92.05 for ^13^C_6_-glucose was used for unambiguous identification. In both cases, the average signal intensity for the ROI of the characteristic fragment was generated in SCiLS from the respective tandem MS data. The experiment was performed using five MI animals and three healthy controls. Two MI animals were excluded from the study because no increase in blood glucose level could be confirmed after ^13^C-labeled glucose administration.

#### 2.12.2 LA-ICP-MS

Gd elemental maps of the thin tissue sections were recorded using a laser ablation system model ImageBio266 (Elemental Scientific Lasers, Bozeman, Montana) coupled with a triple quadrupole-based iCAP TQ ICP-MS (Thermo Scientific, Bremen, Germany). Ablation of the samples was performed at a laser spot size of 15 µm. The triple quadrupole was operated in the oxygen mode. The phosphorus and gadolinium were measured as ^31^P^16^O^+^ and ^158^Gd^16^O^+^, respectively. For spatially resolved quantification of Gd, matrix-matched gelatin standards were produced in a concentration range of 5–500 µg g^-1^ Gd using aqueous GdCl_3_ solution and 10% gelatin (w/w). Standards were homogenized at 45 °C and cryosectioned into 10 µm thin sections using a cryotome (Cryostar NX70, Thermo Scientific, Bremen, Germany). Standard thin sections of gelatin were thaw-mounted on glass slides, and analyzed under identical measurement conditions as the tissue samples. Data evaluation and visualization were carried out using the in-house developed software Imajar (by Robin Schmid).

## 3 Results

### 3.1 D-glucose as a biodegradable contrast agent for MI detection using glucoCEST MRI

**Fig. 2:**
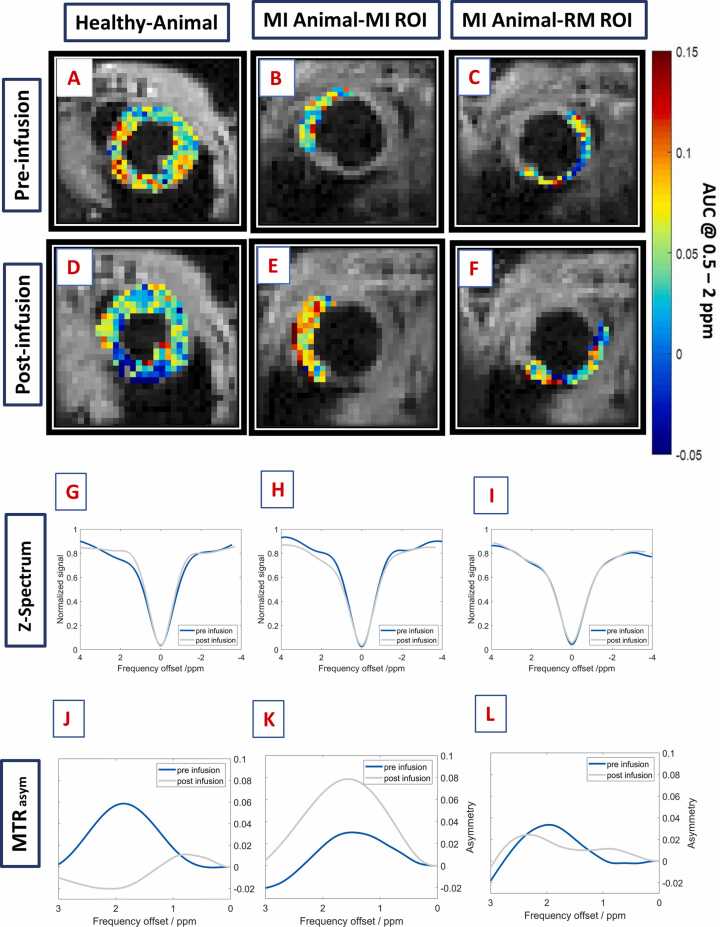
*In vivo* cardiac glucoCEST MTR_asym_ contrast. Exemplary *in vivo* cardiac **(A-F)** glucoCEST MTR_asym_ maps (frequency range of 0.5 to 2 ppm) and corresponding **(G-I)** Z- and **(J-L)** MTR_asym_- spectra of healthy myocardium, MI (Day 7), and RM pre- and postglucose infusion. In the infarcted region, increased MTR_asym_ values are measured in the frequency range of 0.5 to 2 ppm after glucose infusion, leading to an enhanced glucoCEST contrast. *glucoCEST* glucose-weighted chemical exchange saturation transfer, *MTR*_*asym*_ magnetization transfer ratio asymmetry, *MI* myocardial infarction

Mice undergoing MI were investigated on day 7 post-surgery and compared with healthy control animals using glucoCEST MRI pre- and postglucose infusion. Individual animals showed substantial differences in the corresponding pixel-wise glucoCEST contrast between the three different regions of MI, RM, and healthy myocardium (Fig. 2A–F).

In the corresponding ROI-based Z-spectra, a distinct glucoCEST signal was detected within the MI region (Fig. 2G–I). Closer examination of the individual Z-spectra acquired pre- and postglucose infusion revealed substantial contributions from direct water saturation and macromolecular magnetization transfer effects, which confounded straightforward spectral subtraction and thus impeded direct isolation of the glucoCEST contrast. However, within the MI region, an increase in MTR_asym_ values in the 0.5–2 ppm range was observed following glucose administration, whereas a decrease was evident in healthy myocardial tissue and minimal changes were detected in the RM (Fig. 2J–L).

**Fig. 3:**
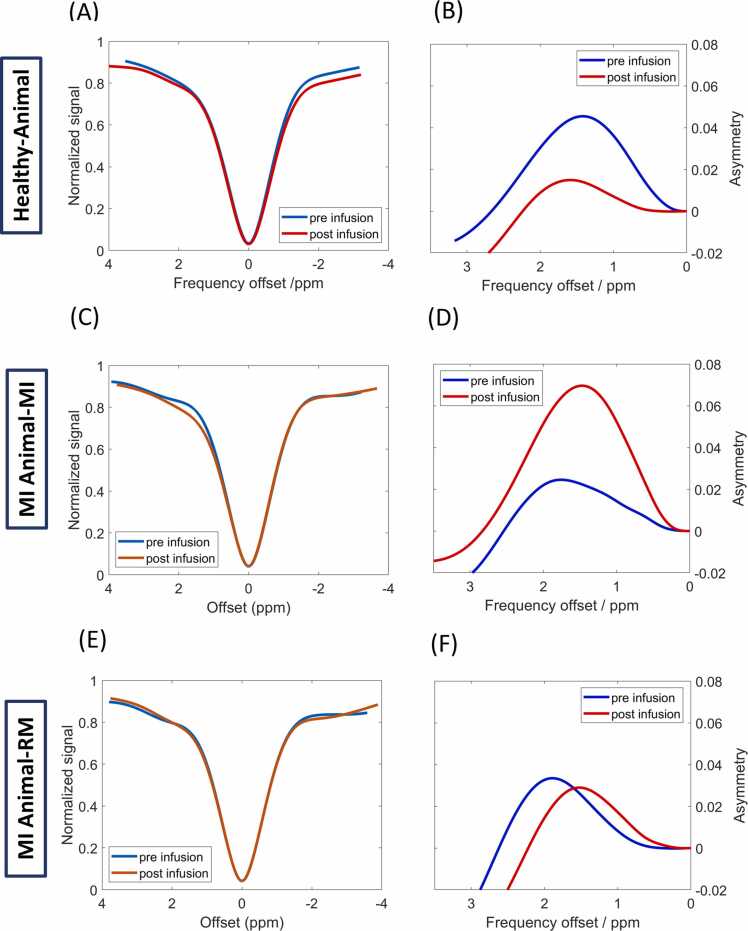
Group analysis of *in vivo* cardiac glucoCEST and MTR_asym_- spectra. GlucoCEST and MTR_asym_-spectra averaged over all healthy animals (n = 8) and animals with MI (n = 6) pre- (blue) and post-(red) glucose infusion. Illustration of spectra from ROIs of **(A, B)** healthy myocardium, **(C, D)** MI, and **(E, F)** RM. *MTR*_*asym*_ magnetization transfer ratio asymmetry, *glucoCEST* glucose-weighted chemical exchange saturation transfer, *MI* myocardial infarction, *ROI* region of interest, *RM* remote myocardium

**Fig. 4:**
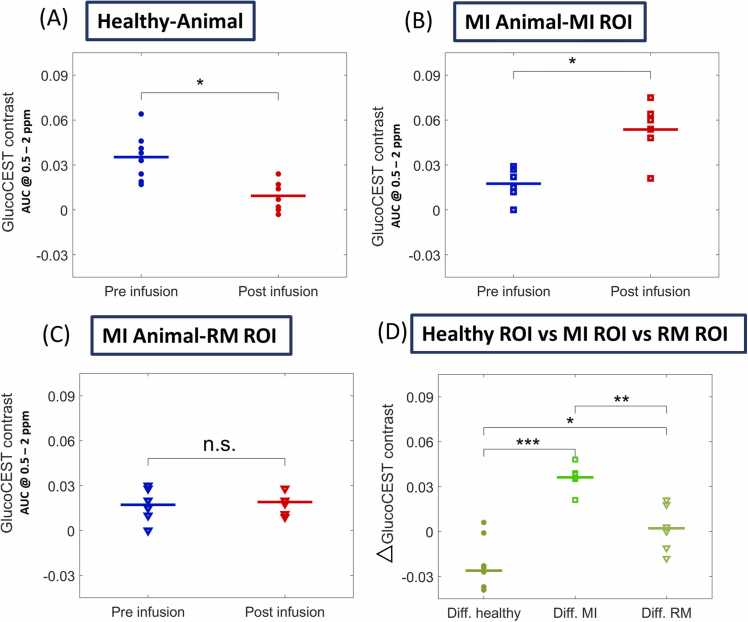
Statistical group analysis of glucoCEST contrast. Statistical analysis of glucoCEST MTR_asym_ values (frequency range of 0.5 to 2 ppm) calculated across all animals and corresponding ROIs pre- (blue) and post- (red) glucose infusion: **(A)** healthy (n = 8), **(B)** MI (Day 7; n = 6) and **(C)** RM region. **(D)** Statistical analysis of the Δ glucoCEST contrast, calculated as difference in contrast between post- and preglucose administration, confirmed significant differences between healthy, MI, and RM ROIs. Each symbol indicates the individual glucoCEST contrast/ Δ glucoCEST contrast value for each animal, and lines indicate the mean group value. P-values were calculated based on the Wilcoxon signed-rank or Mann-Whitney U-test (*p<0.05, **p<0.01, ***p<0.001). *MTR*_*asym*_ magnetization transfer ratio asymmetry, *glucoCEST* glucose-weighted CEST, *MI* myocardial infarction, *ROI* region of interest, *RM* remote myocardium

Group analysis of Z- (Fig. 3A, C, E) and corresponding MTR_asym__-_ (Fig. 3B, D, F) spectra confirmed these findings. Specifically, in healthy myocardium, the mean MTR_asym_ value after glucose infusion significantly decreased from 0.035 ± 0.014 to 0.009 ± 0.009 (p<0.01) (Fig. 4A). Conversely, the mean MTR_asym_ value was significantly increased in the MI region from 0.017 ± 0.010 to 0.054 ± 0.017 (p<0.05) (Fig. 4B), whereas in the RM of infarcted animals, the mean MTR_asym_ value remained nearly unchanged at 0.017 ± 0.010 *vs.* 0.019 ± 0.007 (Fig. 4C). The average change in MTR_asym_ values pre- and postglucose infusion significantly differed among the three ROIs (MI, RM, and healthy myocardium; Fig. 4D). Notably, the baseline mean MTR_asym_ values prior to glucose infusion could already significantly distinguish between MI and healthy animals (p<0.05), showing a lower glucoCEST contrast in the MI and RM regions. However, no significant differences were observed between the infarcted zones and the RM.

To assess potential contamination of the calculated glucoCEST contrast by direct water saturation and T₂ effects, we validated our results using a Lorentzian difference analysis. This approach involved subtracting a mono-Lorentzian fit of the water peak from the Z-spectra and analyzing the resulting residuals (Supplemental Fig. 6). Quantification of the AUC within the frequency range of 0.5–2.0 ppm in the residual spectra confirmed the MTR_asym_ findings, showing the highest glucoCEST contrast in the MI region (Supplemental Figs. 7 and 8). Furthermore, the full width at half maximum (FWHM) of the Lorentzian component sensitive to T₂ effects did not exhibit significant differences between pre- and postglucose infusion in any of the groups (Supplemental Fig. 9).

### 3.2 MRI relaxometry in myocardial infarction

**Fig. 5:**
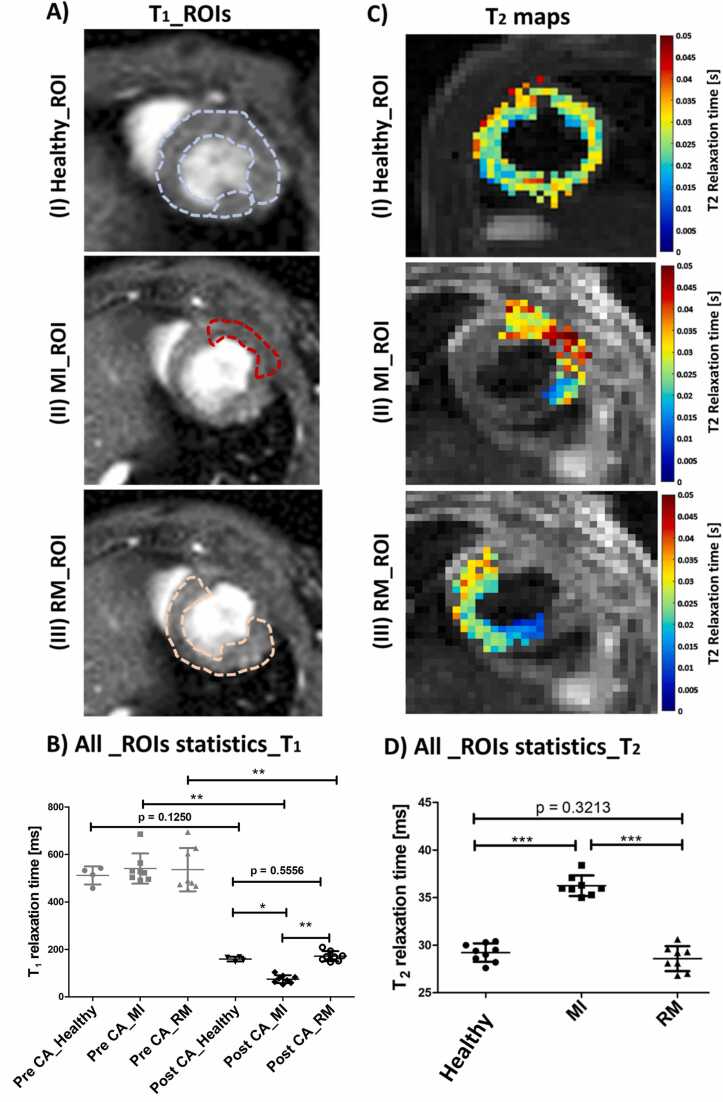
Quantitative analysis of T_1_ and T_2_ relaxation times. **(A)** Representative T_1_-weighted images for three distinct conditions: healthy, MI (Day 7), and RM, along with delineated ROIs. **(B)** Statistical analysis of T_1_ relaxation times for both healthy and MI animals, calculated across all animals and defined ROIs pre- and post-administration of the Gd-based contrast agent (CA) gadofosveset (healthy, n = 4; post-MI, n = 8). **(C)** Representative T_2_ relaxation time maps for the same three conditions with delineated ROIs. **(D)** Corresponding statistical analysis of T_2_ relaxation times for both healthy and MI animals (healthy, n = 9; post-MI, n = 8). Each symbol indicates the individual T_1_/ T_2_ relaxation times for each animal. The midlines represent the mean group values, while the small lines indicate the standard deviations. Significant differences between the groups were determined using the Wilcoxon signed-rank or Mann-Whitney U-Test, with p-values indicating the level of significance (*p<0.05, **p<0.01, ***p<0.001). *MTR*_*asym*_ magnetization transfer ratio asymmetry, *glucoCEST* glucose-weighted CEST, *MI* myocardial infarction, *ROI* region of interest, *RM* remote myocardium

Following the glucoCEST experiment, LGE MRI was conducted to validate the glucoCEST findings and to distinguish between different myocardial tissue states *in vivo*. The MI zone was identified using gadofosveset, leading to an accumulation of Gd-chelates and bright image contrast in the neovascularized and remodeled tissue of the infarcted region in comparison to healthy tissue and RM (Supplemental Fig. 6A, B). Distinct patterns of Gd distribution induced significant changes in the T_1_ relaxation times across the three different myocardial regions following Gd administration (Fig. 5A (I, II, III), B). Specifically, the MI regions exhibited significantly decreased T_1_ values (pre-CA 556 ± 78 ms and post-CA 80 ± 17 ms, p=0.0079) compared with healthy tissue (pre-CA 512 ± 38 ms vs. post-CA 159 ± 10 ms, p = 0.0286) and RM (pre-CA 548 ± 105 and post-CA 169 ± 25, p = 0.0079; Fig. 5B). In addition, insight into edema formation in the myocardium was obtained by calculating T_2_ relaxation times without the use of contrast agents showing significantly increased T_2_ values in MI regions (36.3 ± 1.1 ms) in contrast to healthy tissue (29.2 ± 1.0 ms) and RM (28.6 ± 1.3 ms) (p≤0.0001) (Fig. 5C (I, II, III), D).

### 3.3 GlucoCEST and LGE MRI lead to comparabl identification of MI regions

**Fig. 6:**
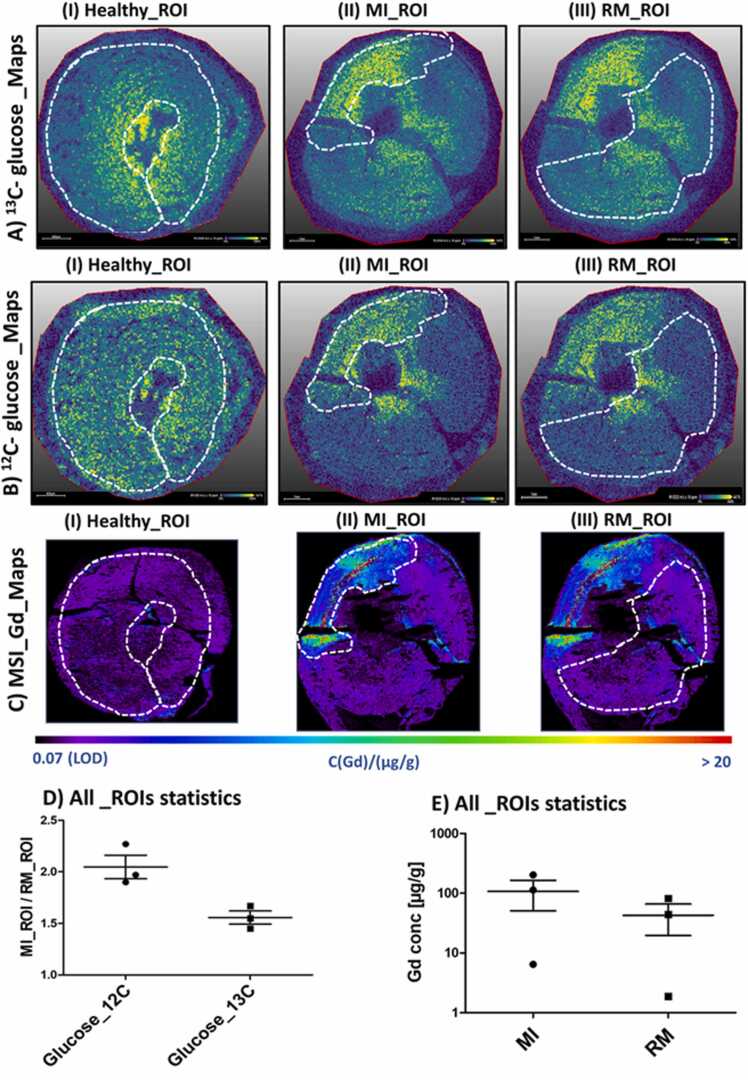
MALDI-2-MSI of ^13^C-labeled and ^12^C-unlabeled glucose as well as LA-ICP-MSI of Gd distributions from myocardial tissue sections. Exemplary illustrations of **(A)**^13^C-labeled and **(B)**^12^C-unlabeled glucose maps as well as **(C)** Gd distributions in healthy myocardium (I), MI (Day 7; II), RM (III). **(D)** Dot plots depict the ratio of glucose accumulation in MI and RM ROIs for both ^12^C-unlabeled (endogenous glucose) and ^13^C-labeled glucose (exogenous glucose) (MI animals n = 3). Values were substantially increased in the MI region for both glucose derivatives. **(E)** In addition, increased Gd concentration was detected in the MI regions compared to RM (MI animals n = 3). Each symbol indicates individual animals. The midlines represent the mean group values, while the small lines indicate the standard deviations. *MALDI-2-MSI* matrix-assisted laser desorption/ionization mass spectrometry imaging with laser post-ionization, *LA-ICP-MSI* laser ablation inductively coupled plasma mass spectrometry imaging, *MI* myocardial infarction, *ROI* region of interest, *RM* remote myocardium

To evaluate the potential of GlucoCEST MRI compared to LGE contrast for distinguishing between MI and RM regions as well as healthy myocardium, mass spectrometric analysis was conducted using MALDI-MS and LA-ICP-MS with ^13^C-labeled D-glucose as the contrast agent instead of natural D-glucose. While MALDI-MSI offers an assessment of the relative intensities of ^13^C- and ^12^C-glucose between different ROIs, LA-ICP-MSI provides a comprehensive quantitative analysis of Gd accumulation in different regions of the myocardium. Accumulation of ^13^C-glucose was observed in the MI region, while levels remained inconspicuous in the RM and healthy myocardium (Fig. 6A I–III). Interestingly, despite the administration of ^13^C-glucose, the signal intensity of ^12^C-glucose also increased in the infarcted region (Fig. 6B II). The accumulation of both ^13^C-glucose and ^12^C-glucose differed substantially between the MI and RM regions (Supplemental Fig. 10A).

ROI-based analysis resulted in increased ^13^C-glucose and ^12^C-glucose intensity ratios of MI to RM in the MI group, with values of 1.557 ± 0.110 and 2.047 ± 0.197, respectively (Fig. 6D). LA-ICP-MS confirmed the LGE results (Fig. 5B), providing a comprehensive quantitative analysis of Gd accumulation in different myocardial regions (Fig. 6C), and revealed higher Gd concentrations in the infarcted region compared to RM (Fig. 6C, E). All individual maps of the healthy and MI animals exhibited significant differences in Gd distribution (Supplemental Fig. 10B).

### 3.4 Border Zones of MI and RM

**Fig. 7:**
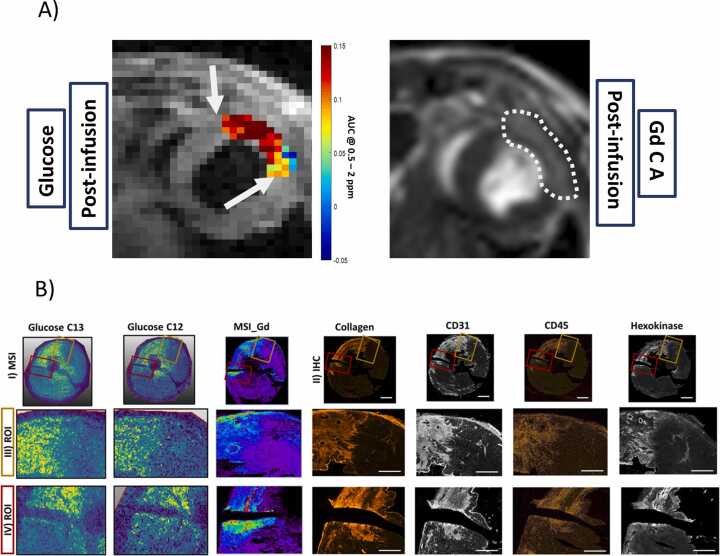
Visualization of the border zones between MI and RM *in vivo* and *ex vivo*. **(A)***In vivo* glucoCEST maps in comparison with LGE. Border zones with reduced glucoCEST contrast are highlighted by white arrows **(B)** MSI maps and immunohistochemistry images at the border zones between MI and RM regions. Visual comparison of **(I)** MSI maps of glucose (both ^13^C-labeled and ^12^C-unlabeled glucose) and Gd with **II)** immunohistochemistry images of collagen 1, CD31, CD45 and hexokinase 1 confirmed good agreement at the two border zones **(III, IV)** between MI and RM regions (red and yellow boxes). Scale bars are 1 mm for the overview tile scans and 500 µm for the ROIs. *MI* myocardial infarction, *RM* remote myocardium, *CEST* chemical exchange saturation transfer, *LGE* late gadolinium enhancement, *MSI* mass spectrometry imaging, *Gd* Gadolinium

In the *in vivo* glucoCEST MRI experiments, MI regions selected for quantitative analysis were defined based on LGE contrast. However, when examining the border zones between the MI and RM in the glucoCEST maps postglucose administration, a marked reduction in CEST contrast values was detected (see arrows in Fig. 7A).

To further explore these observations, *ex vivo* comparisons were performed between the ¹²C/¹³C-glucose distribution maps and MSI Gd data (Fig. 7B). As described in the previous section, a substantial spatial overlap between glucose- and Gd-derived maps was evident; nevertheless, notable discrepancies emerged specifically within the border zones.

While Gd distributions showed a strong correlation with the enhanced expression of tissue remodeling and inflammatory biomarkers—including collagen I (Col 1), CD31, CD45, and hexokinase 1 (HK1), as confirmed by IHC—the agreement between the ¹²C/¹³C-glucose maps and these biomarker expression patterns was less pronounced in the same regions.

This reduced concordance between glucose-related contrasts and Gd enhancement in the MI border zones supports the low *in vivo* glucoCEST contrast values, indicating that glucose accumulation and metabolism in these transitional regions differ substantially from those in the infarct core and RM. A detailed description of the underlying cellular and vascular interactions and structural reorganization within the MI and RM zones, compared with healthy myocardium, is provided in the Supplementary Information (Supplemental Fig. 11A–F; Supplemental Results 1).

## 4 Discussion

This study explored the potential of *in vivo* CEST MRI using natural D-glucose as a biodegradable contrast agent to identify and evaluate MI in mice on day 7 post-induction. GlucoCEST MRI effectively discriminated between the MI region, RM, and the healthy myocardium. This study provides comprehensive experimental insights into the mechanisms of *in vivo* glucoCEST MRI mapping in both MI and RM regions, achieved through the distinction between exogenously administered glucose and endogenous glucose using ^13^C-labeled glucose as a contrast agent. The results from these *in vivo* experiments are consistent with LGE MRI, as well as with *ex vivo* findings from MSI for ^13^C/^12^C-glucose visualization, Gd quantification, and IHC within the MI, RM, and healthy regions.

Our findings revealed a significant increase in glucoCEST contrast in the MI region seven days post-MI following the infusion of natural biodegradable D-glucose (Fig. 4B). Conversely, we observed decreased and unchanged glucoCEST contrasts in the healthy and RM regions, respectively (Fig. 4A, C). An additional analysis of glucose accumulation was conducted using MSI, with the application of ^13^C-labeled glucose. Interestingly, both ^13^C-glucose and ^12^C-glucose MSI maps revealed substantially higher glucose accumulation in the MI region than in the RM region (Fig. 6A II, III). These results suggest that a different underlying mechanism is responsible for the signal generation in glucoCEST MRI in comparison to ^18^F-FDG PET, which commonly results in reduced signal intensities in zones of infarction[41]. Whereas ^18^F-FDG-PET detects intracellularly accumulated phosphorylated fluorine-18 glucose intermediates[41], according to the metabolic turnover, the glucoCEST signal is mainly derived from ‘intact’ glucose. Although intracellular hydroxyl protons of glycolytic intermediates such as glucose-6-phosphate, fructose-6-phosphate, and fructose-1,6-biphosphate can be detected by glucoCEST, their concentrations are extremely low and they are rapidly converted to lactate and pyruvate [21], [42]. Given that the glucose concentration administered in the glucoCEST experiment is significantly higher than that of ^18^F-FDG tracers, the observed enhancement of glucose accumulation in the MI region is likely driven by both increased myocardial metabolic activity and perfusion-related factors such as blood volume, vascular leakage, and extracellular space dynamics [43], [44]^,^[6].

Consequently, a combination of circulatory function, glucose washout, and molecular factors -such as extracellular pH, ionic strength, and microenvironmental influences affecting glucose dynamics and chemical exchange—may contribute to the observed effect [45], [46]. The pronounced glucoCEST contrast detected in the MI region likely arises from glucose accumulation within the acidic extracellular space due to impaired cellular uptake, where the reduced pH slows proton exchange kinetics, thereby enhancing the glucoCEST signal [19]. Our glucoCEST results, both pre- and postglucose infusion, clearly reflected this condition. Pre-glucose infusion, the glucoCEST contrast in the MI and RM regions of MI animals showed reduced values compared to healthy controls, indicating either increased glucose utilization or decreased glucose availability, as discussed in previous literature [47]. In MI regions, the well-established metabolic shift from predominantly fatty acid oxidation toward increased glycolysis results in glucose becoming the primary energy source. This metabolic adaptation is thought to support cellular survival and tissue repair under conditions of oxidative stress and impaired perfusion.

However, postglucose infusion, differential glucoCEST contrast occurred between the MI and RM regions due to cardiac tissue remodeling, including fibrosis, immune cell infiltration, angiogenesis, and vascular leakage in the transmural MI region [48], [49], [50], [51], [52], as demonstrated by our IHC results (Supplemental Fig. 11A–F, Fig. 7). This suggests that perfusion-related properties could be a major source of glucose accumulation in the extracellular space [53], particularly given the high expression levels of HK1 observed in the MI region in our IHC results, which might arise from metabolically active immune cells and endothelial cells that consume glucose. In addition, the observed increased ^12^C-unlabeled glucose in the MI region after ^13^C-labeled glucose administration indicates competitive glucose utilization between the two isotopes.

In the border zones, however, glucose accumulation was markedly lower than Gd uptake (Fig. 6, Fig. 7). While Gd enhancement primarily reflects tissue remodeling processes that alter perfusion—such as fibrosis, necrosis, and expansion of the extracellular space—glucose uptake depends not only on vascular properties but also on active transport (e.g., GLUT1 and GLUT4) and metabolic turnover [54], [55], [56]. In these regions, alterations in metabolism of viable immune and endothelial cells likely contribute to the observed discrepancies between Gd and ^13^C/^12^C-glucose maps. Further studies will be necessary to evaluate the differential diagnostic potential of glucoCEST contrast for delineating myocardial border zones.

The observed reduction in glucoCEST contrast in healthy myocardium following glucose infusion, compared to baseline, may appear paradoxical. However, it is well established that elevated blood glucose and insulin levels alter myocardial substrate preference and induce vasodilation [57], [58]. These effects can enhance both myocardial glucose metabolism and perfusion, potentially contributing to a faster washout of glucose and, consequently, reduced glucoCEST signal. This interpretation is further supported by the comparable signal distribution curves of unlabeled (¹²C) and labeled (¹³C) glucose observed in MSI, which indicate consistent glucose handling within the myocardium.

Based on our findings, the clinical use of glucoCEST MRI may have a significant impact on the long-term management of patients with MI. While echocardiography is the primary diagnostic imaging tool in the acute stage of MI owing to its easy accessibility and effectiveness, patients in the post-acute phase benefit from MRI and PET [59], [60]. In particular, LGE is clinically established to precisely identify the location and extent of the infarcted region, and is capable of differentiating between viable and non-viable myocardium [6], [7]. Since LGE and glucoCEST MRI results overlapped over a broad region (Fig. 7), glucoCEST MRI could serve as a kidney-friendly alternative to LGE in patients with renal disease. The feasibility of translating glucoCEST MRI using natural D-glucose in clinical settings has already been demonstrated in several proof-of-concept studies [29], [30], [31]. A glucose-infusion protocol similar to that described in (Supplemental Fig. 1) might be adapted to human physiology to achieve a stable hyperglycemic blood glucose condition. Recent advancements in cardiac CEST MRI, as reported by Schache et al. [33], additionally facilitate the clinical translation of cardiac CEST techniques by enabling their use without the need for sophisticated trigger and gating strategies.

## 5 Limitations

While the proposed glucoCEST approach shows clear potential for the detection of MI, several limitations of the present study should be considered.

CEST MRI in general is characterized by relatively low contrast and high sensitivity to motion as well as to B_0_ and B_1_ field inhomogeneities. While B_0_ correction was applied in our analysis, B_1_ correction was not implemented in the current processing pipeline. In addition, the saturation pulse used for CEST acquisition was relatively short. As reported by Schache et al. [33], short pulses are susceptible to Rabi oscillations, which can affect quantification accuracy. Although a dedicated and optimized CEST analysis pipeline was established, minor contamination artifacts may still persist. It also remains uncertain whether the short saturation pulses used here will be sufficient to achieve robust contrast in future patient studies at lower field strengths. However, since the human cardiac cycle is longer, the required saturation pulses could be easily adjusted and extended accordingly. Given the fast exchange between glucose hydroxyl protons and water protons, higher B₁ amplitudes may additionally be beneficial and improve the detection limit, as reported for tumor xenografts by Zaiss et al. [45].

Another limitation arises from the murine MI model used. In this study, MI was induced in mice *via* left thoracotomy and permanent ligation of the left coronary artery, a procedure known to produce variability in infarct transmurality depending on surgical precision. This variability can influence both perfusion and tissue remodeling in the infarcted region. In addition, the use of isoflurane anesthesia may have affected glucose metabolism, potentially influencing the observed glucoCEST contrast. Finally, the relatively small sample size of six animals limits the statistical power and generalizability of the results.

Future studies will therefore focus on further technical validation and biological evaluation of the proposed glucoCEST methodology. In particular, detailed investigations at lower field strengths and in different MI models, including longitudinal designs, are planned to improve accuracy, reproducibility, and translational relevance.

## 6 Conclusion

In this study, an *in vivo* cardiac glucoCEST MRI method that utilizes natural biodegradable D-glucose as a contrast agent was used to identify the infarction region, allowing for effective differentiation between the MI region, RM, and healthy myocardium. Although glucoCEST MRI is still in its infancy in clinical practice, this study may serve as a starting point for advancing cardiac glucoCEST MRI techniques for MI follow-up, complex cases, and long-term patient management.

## Funding

This work was supported by the Deutsche Forschungsgemeinschaft (GermanResearch Foundation) Project ID 406818964 to Cornelius Faber and Verena Hoerr; DFG–CRC1450–431460824 (project C05) to Verena Hoerr; Project ID 249895739 to Cornelius Faber, SFB1009–194468054 (project A09), and Core unit PIX of the interdisciplinary center for clinical research Münster.

## Author contributions

**Ajay Peddi:** Conceptualization, Data curation, Formal analysis, Investigation, Methodology, Validation, Visualization, Writing - original draft, Writing - review & editing. **Daniel Schache:** Formal analysis, Software, Validation, Visualization, Writing - review & editing. **Chris Lippe:** Formal analysis, Software, Validation, Visualization, Writing - review & editing. **Sai Kiran Reddy Samawar**: Data curation, Methodology, Writing - review & editing. **Michael Kuhlmann:** Methodology, Writing - review & editing. **Peter Niehaus**: Data curation, Formal analysis, Methodology, Writing - review & editing. **Jens Soltwisch**: Data curation, Formal analysis, Methodology, Validation, Writing - review & editing. **Emily Hoffmann:** Methodology, Writing - review & editing. **Ali Nahardani**: Formal analysis, Writing - review & editing. **Stephan Niland:** Methodology, Writing - review & editing. **Noelia Alonso Gonzalez:** Methodology, Conceptualization. **Klaus Dreisewerd:** Methodology, Resources. **Uwe Karst**: Methodology, Resources. **Lydia Sorokin:** Methodology, Resources, Validation. **Michael Schaefers**: Methodology, Resources. **Moritz Wildgruber**: Conceptualization, Funding acquisition, Writing - review & editing. **Cornelius Faber:** Conceptualization, Formal analysis, Funding acquisition, Project administration, Resources, Supervision, Validation, Writing - review & editing. **Verena Hoerr**: Conceptualization, Funding acquisition, Investigation, Methodology, Data curation, Formal analysis, Project administration, Resources, Software, Supervision, Validation, Writing - original draft, Writing - review & editing. All authors have read and agreed to the published version of the manuscript.

## Ethics approval and consent

All experimental and animal husbandry procedures were conducted strictly in accordance with the German Animal Welfare Act and local animal welfare guidelines and had received full approval from the Landesamt für Natur, Umwelt und Verbraucherschutz NRW (Protocol No. 81–02.04.2019. A169).

## Consent for publication

The manuscript is approved by all authors for publication.

## Declaration of competing interests

The authors declare that they have no known competing financial interests or personal relationships that could have appeared to influence the work reported in this paper.

## Footnotes

Appendix A Supplementary data associated with this article can be found in the online version at doi:10.1016/j.jocmr.2025.102667.
