# Supplementary material for: Characterization of myocardial infarction by in vivo chemical exchange saturation transfer magnetic resonance imaging using natural D-glucose
Source: J Cardiovasc Magn Reson. 2025 Nov 30;28(1):102667. doi: 10.1016/j.jocmr.2025.102667 (PMC12808883; doi:10.1016/j.jocmr.2025.102667)
Supplement: Supplementary file 1 — Supplementary material [file mmc1.zip › Supplemental Figure Legends.docx]

**Supplemental Figure Legends**

**Supplemental Figure 1.** **Different glucose administration protocols applied to healthy and MI animals: (A)** i.p. and **(B)** i.v. administration of subsequent injections of 1 M and 1.5 M glucose solutions with a time interval of 25 min (4.25 µl/g body weight). While the i.p. route resulted in an elevated, constant blood glucose level 45 min post-injection, the glucose concentration decreased quickly after i.v. administration and decayed briefly thereafter. **(C)** i.p. administration of only one single bolus injection of 1 M glucose solution (4.25 µl/g body weight), showing rapidly declined blood glucose.  **(D)** Group analysis: All healthy (n=8) and MI (Day 7 n=6) animals included in the study confirmed significantly elevated glucose levels 60 min after the second glucose bolus injection, measured following the MRI. Neither in healthy (n=3) nor in MI (n=3) animals, the **(E)** T_1_ and **(F)** T_2_ relaxation time was statistically impacted by the i.p. administration of glucose. Each symbol indicates the individual T_1_ and T_2_ relaxation times for each animal. The midlines represent the mean group values while the small lines indicate the standard deviations. P-values were calculated using the Wilcoxon signed-rank or Mann-Whitney U-Test (*p<0.05, **p<0.01, ***p<0.001).

**Supplemental Figure** **2.** **Schematic illustration of cardiac glucoCEST MRI data analysis pipeline**.

**Supplemental Figure 3. Effect of repetition time on glucoCEST contrast in vitro.** Z (blue) and MTRasym spectra (red) of a 75 mM glucose phantom dissolved in PBS acquired using a short saturation pulse (140 ms) at varying repetition times: **(A**) 386 ms, **(B)** 1000 ms, **(C)** 5000 ms, **(D)** 10000 ms.

**Supplemental Figure 4. Experimental validation of glucoCEST contrast in vitro and in vivo. A)** MTRasym contrast (frequency range of 0.5 to 2 ppm) of a glucose concentration series (1, 2.5, 5, 7.5, 10, 15, 20, 30, 40, 45, 60, 75 mM glucose in PBS) acquired with a 140 ms CEST pulse and 386 ms repetition time, calculated from analyzed Z spectra after DOSE filtering, showed a strong linear correlation with glucose concentration (R² > 0.99). The measurements were performed at pH 7.4 and at room temperature (18–20 °C), monitored by a temperature probe. **B)** Bloch–McConnell simulation of MTR_asym_ contrast before and after glucose infusion, based on a cardiac tissue model containing the following proton pools: water, MT, hydroxyl, amine, and amide.

**Supplemental Figure 5. T_1_ relaxation times in MI pre- and post-Gd-CA:** A series of T_1_-weighted MR images of an animal with MI (Day 7) is illustrated with variable flip angles **(A)** pre and **(B)** post-administration of the Gd-based contrast agent (Gd-CA) gadofosveset. Accumulation of Gd-CA in the MI region resulted in pronounced signal enhancement (orange arrow). **(C)** ROI-based T_1_ relaxation times were calculated by fitting the signal intensity to the Ernst equation.

**Supplemental Figure 6. In vivo Lorentzian Difference Analysis.** Exemplary *in vivo* cardiac Z-spectra, corresponding Mono-Lorentzian fit to the water peak and residuals for (A) healthy myocardium, (B) MI (Day 7), and (C) RM shown pre- and post-glucose infusion. In the infarcted region, pronounced residual signals are observed in the frequency range of 0.5 to 2 ppm after glucose infusion, leading to an enhanced glucoCEST contrast.

**Supplemental Figure 7. In vivo analysis of Z-spectral residuals.** Exemplary glucoCEST contrast in MI ROI, calculated as the AUC of the Z-spectral residuals in the frequency range of 0.5 to 2 ppm, shown (A) pre- and (B) post-glucose infusion.

**Supplemental Figure 8: Statistical group analysis of glucoCEST contrast.** Statistical analysis of the glucoCEST residual AUC in the frequency range of 0.5 to 2 ppm calculated across all animals and corresponding ROIs, pre- and post-glucose infusion: **(A)** healthy (n=8), **(B)** MI (Day 7; n=6) and **(C)** RM region. **(D)** Statistical analysis of the Δ glucoCEST contrast, calculated as difference in glucoCEST residual AUC between post- and pre-glucose administration, confirmed significant differences between healthy, MI, and RM ROIs. Each symbol indicates the individual glucoCEST contrast/ Δ glucoCEST contrast value for each animal, and lines indicate the mean group value. P-values were calculated using the Wilcoxon signed-rank or Mann-Whitney U-Test (*p<0.05, **p<0.01, ***p<0.001).

**Supplemental Figure 9. Statistical group analysis of the Mono-Lorentzian fits to the water peak.** Statistical analysis of the FWHM values calculated across all animals and corresponding ROIs pre- and post-glucose infusion: **(A)** healthy (n=8), **(B)** MI (Day 7; n=6) and **(C)** RM region. P-values were calculated using the Wilcoxon signed-rank Test (*p<0.05, **p<0.01, ***p<0.001).

**Supplemental Figure 10. Pixel-wise Gd and glucose distributions determined by MSI:** **(A)** Pixel-wise signal distributions of ^13^C-labeled and ^12^C-unlabeled glucose measured by MSI 60 minutes post-administration of ^13^C-labeled glucose for both **(I-III)** healthy animals (n=3) and **(IV-VI)** those with MI (n=3). For all MI sections, signal intensities of both ^13^C-labeled and ^12^C-unlabeled glucose showed substantially elevated values in comparison to healthy tissue and RM. **(B)** In analogy, quantitative analysis of Gd distributions in healthy (n=1) and MI (n=3) sections following LGE experiments confirmed significantly elevated Gd concentrations in regions of MI in comparison to RM and healthy tissue. The p-values were calculated based on the Mann-Whitney U-Test (*p<0.05, **p<0.01, ***p<0.001).

**Supplemental Figure 11. Immunofluorescence images of healthy and MI (Day 7) mouse heart samples.** **(A-C)** Immunofluorescence staining and confocal microscopy images of mouse heart sections showing extracellular matrix markers, blood vessels, fibroblasts and immune cells in health myocardium, RM and MI tissues: **(A)** collagen 1 (Col 1 - orange), CD31 (white), and DAPI (turquoise), **(B)** CD45 (orange), pan laminin or laminin 111 (white), and PDGFR⍺ (green), **(C)** Isolectin GS-B4 (red) and DAPI (blue). **(D-E)** Immunofluorescence images showing macrophages (F4/80 - orange, CD206 - green), the glucose metabolism marker hexokinase 1 (HK1 - white) and DAPI (turquoise) in healthy myocardium, RM and MI tissues. **(E, F)** Immunofluorescence staining’s show altered expression of gap junction markers **(E)** Cx37 (green) and **(F)** Cx43 (green) in combination with the blood vessel marker CD31 (white) and DAPI (blue) in healthy myocardium, RM and MI regions. Images are representative of n=2 mice per condition and scale bars indicating 25 µm for the merged images and 50 µm for single channel images.
