# Supplementary material for: Characterization of myocardial infarction by in vivo chemical exchange saturation transfer magnetic resonance imaging using natural D-glucose
Source: J Cardiovasc Magn Reson. 2025 Nov 30;28(1):102667. doi: 10.1016/j.jocmr.2025.102667 (PMC12808883; doi:10.1016/j.jocmr.2025.102667)
Supplement: Supplementary file 1 — Supplementary material [file mmc1.zip › Supplemental Results 1.docx]

**Supplemental Results 1.**

**Pathophysiological changes assessed by immunohistochemistry**

To obtain a deeper insight into cellular and vascular interactions and reorganization within the zones of MI and RM in comparison to healthy controls seven days post-induction of MI in mice, microscopic investigations were performed along with immunohistochemistry (IHC) (Supplemental Figure 11A-F). In contrast to healthy myocardium, MI tissue revealed a substantial increase in collagen 1 (Col 1) expression (Supplemental Figure 11A), which is associated with fibrosis and scar formation leading to a loss of meticulous fiber organization. Col 1 staining also exhibited a substantially higher intensity and extent in the infarcted region than in the RM. CD31 staining confirmed a robust vascular network of endothelial cells, particularly within large blood vessels (BVs) in the healthy myocardium. Upon closer inspection of ROIs of higher magnification, a notable presence of Col 1 was observed around large CD31+ BVs, whereas in regions of smaller BVs exhibited considerably lower Col 1 expression. This intricate relationship suggests a nuanced connection between collagen distribution and blood vessel size in the healthy myocardium. However, CD31 staining in the MI region displayed background signals, likely arising from tissue damage and leaky vessels (Supplemental Figure 11AIII). The high-magnification ROI of the infarcted region emphasizes damaged CD31+ BVs, indicating a potential correlation between hypoxia and edema in this specific region. DAPI staining, which confirmed proper nucleus shape and size in the healthy and RM regions, showed varying sizes and shapes in the MI, which suggests hypoxic and microenvironmental alterations, such as edema formation. In addition, besides notable infiltration of inflammatory CD45+ immune cells, high expression of PDGFRα+ fibroblasts were observed in the MI tissue (Supplemental Figure 11B). This may explain the enhanced laminin staining along with increased Col 1 expression compared to RM and healthy myocardium. Laminin, which is found in the extracellular matrix, is associated with pronounced microvessel formation (angiogenesis). Increased neovascularization was also confirmed by strong isolectin GS-B4 patterns in the infarcted region (sub-epi- and endocardial regions), whereas a steady-state baseline vascular network was found in the healthy tissue and RM (Supplemental Figure 11C). Aligned with CD45+ staining, we observed an increase in the number of macrophages in the MI region, as seen from the F4/80+ staining, and CD206+ M2 macrophages (Supplemental Figure 11DIII) were detected at high density in MI regions compared to RM. Increased numbers of CD206+ cells play an anti-inflammatory role, which is potentially crucial for tissue repair. Additionally, metabolic alterations towards abnormal glycolysis were indicated by elevated hexokinase 1 (HK1) staining (Supplemental Figure 11DIII). In contrast, high-magnification images of the RM demonstrated F4/80+ and CD206+ cell distribution and low HK1 expression levels, similar to those in healthy tissues. In the healthy myocardium, concentrated HK1 was only found around large BVs. To study the gap junctions, Connexion 37 (Cx37) and Connexion 43 (Cx43) were investigated. Cx37 colocalized with CD31+ BVs in healthy and RM, whereas in the MI region, Cx37 showed a more widespread distribution, indicating its expression not only in vascular endothelial cells, but also in stromal and infiltrating immune cells (Supplemental Figure 11EIII). Conversely, Cx43 was primarily expressed in cardiomyocytes and absent in the vasculature of healthy tissues and RM. It exhibited downregulation and disorganized expression in the infarcted region, most likely because of massive cardiomyocyte death (Supplemental Figure 11F).
