# Supplementary figures and images for: Characterization of myocardial infarction by in vivo chemical exchange saturation transfer magnetic resonance imaging using natural D-glucose
Source: J Cardiovasc Magn Reson. 2025 Nov 30;28(1):102667. doi: 10.1016/j.jocmr.2025.102667 (PMC12808883; doi:10.1016/j.jocmr.2025.102667)

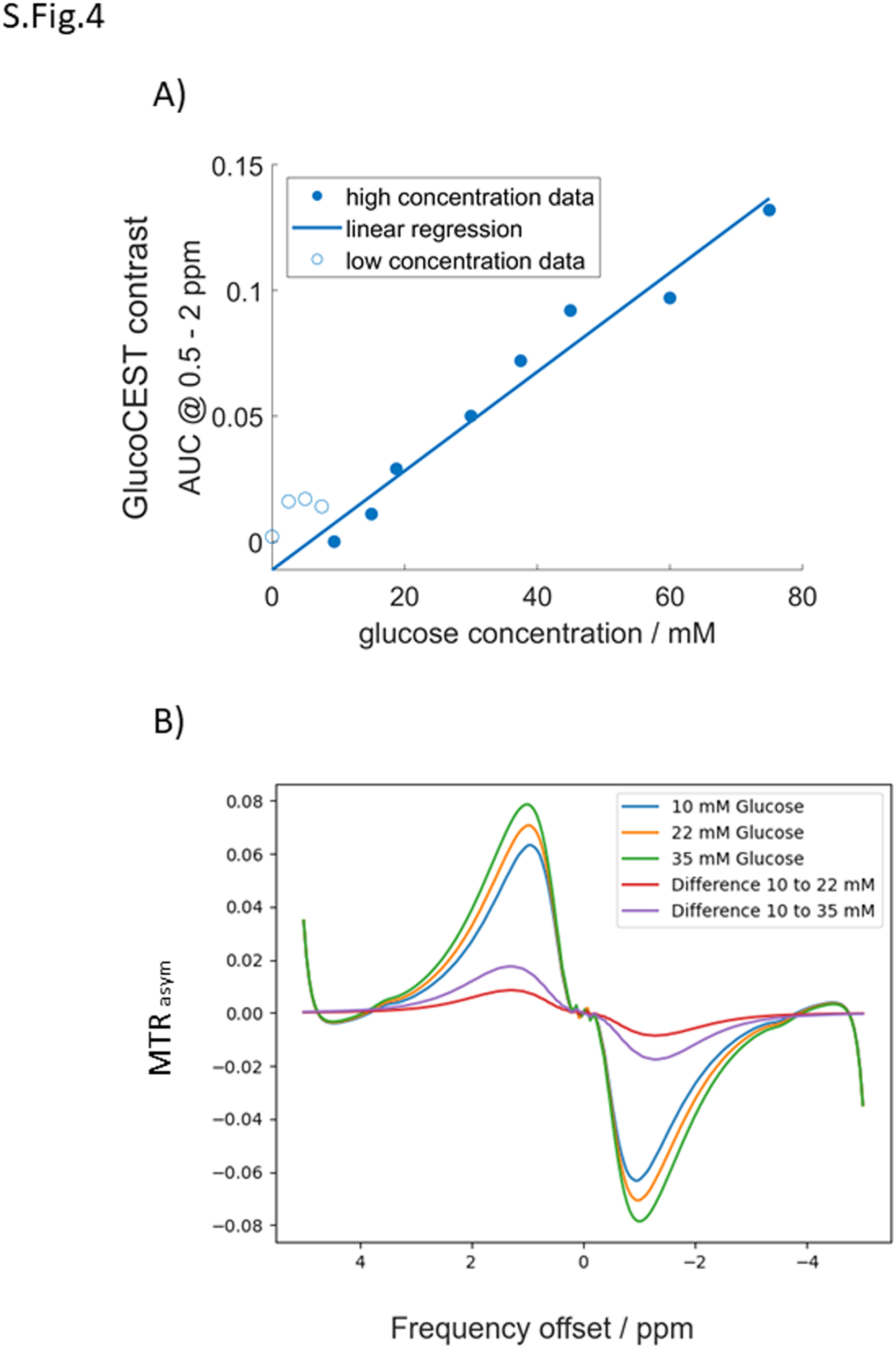

Supplement: Supplementary file 1 — Supplementary material [file mmc1.zip › Supplemental Figure 4 .jpg]

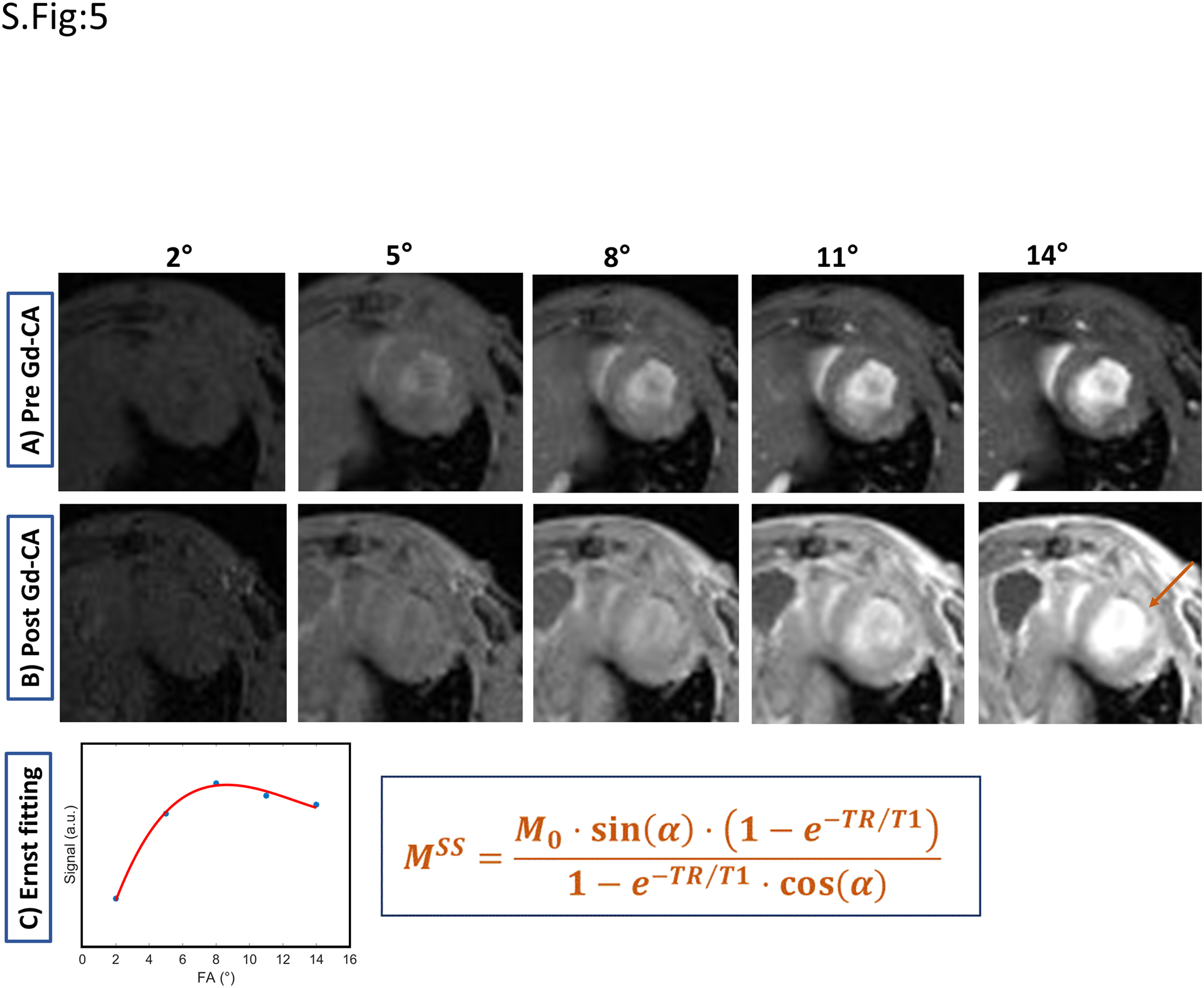

Supplement: Supplementary file 1 — Supplementary material [file mmc1.zip › Supplemental Figure 5.jpg]

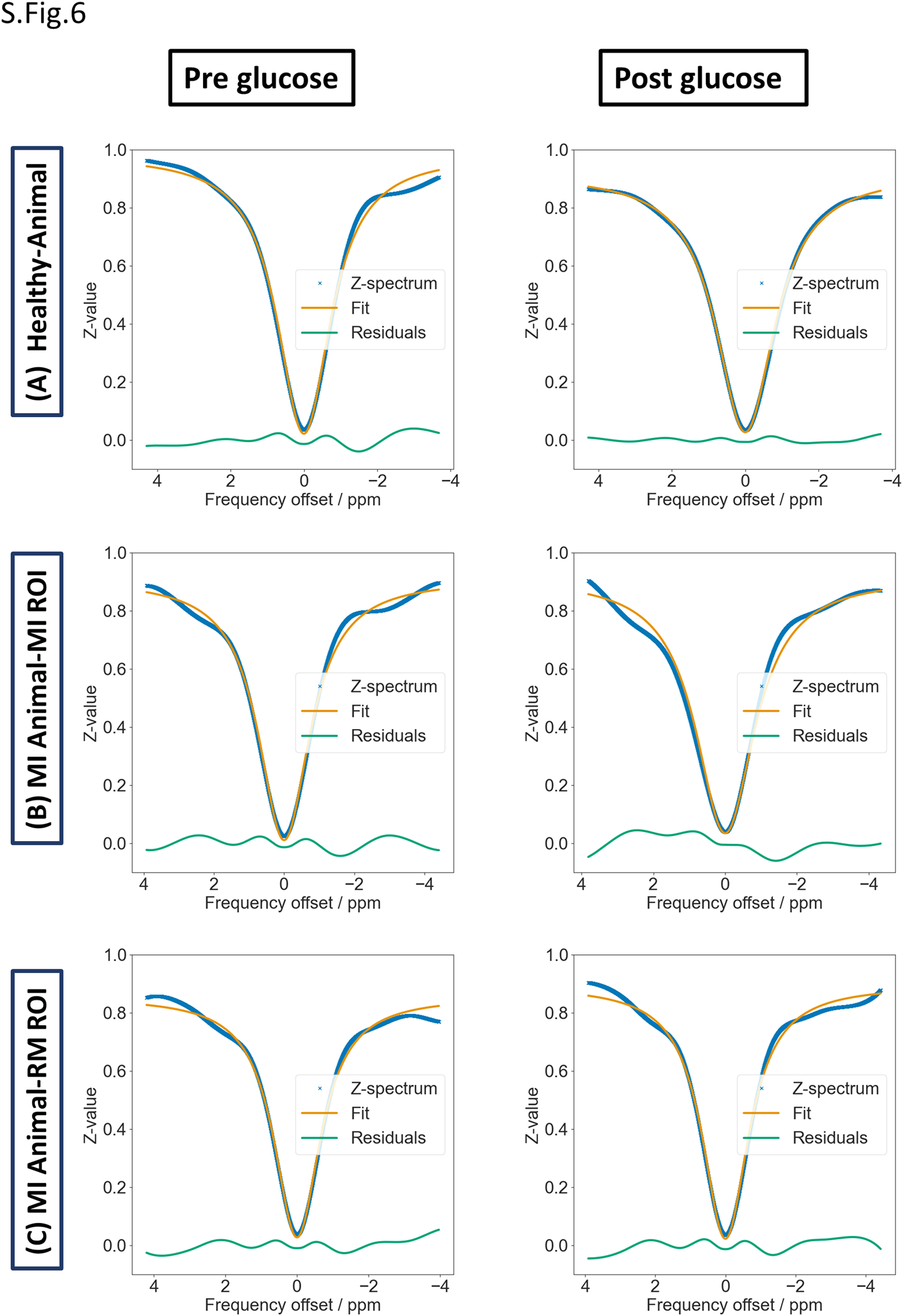

Supplement: Supplementary file 1 — Supplementary material [file mmc1.zip › Supplemental Figure 6.jpg]

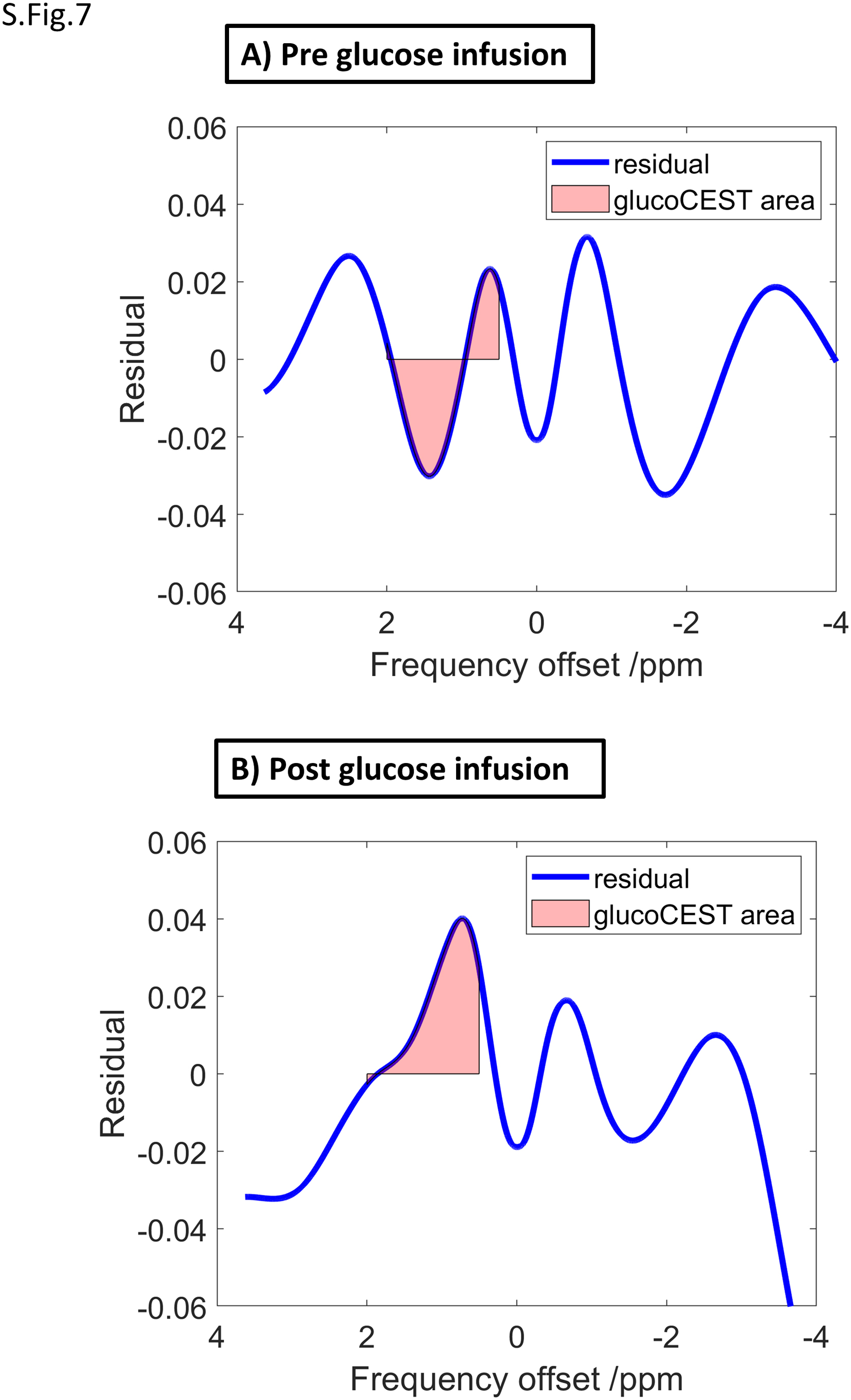

Supplement: Supplementary file 1 — Supplementary material [file mmc1.zip › Supplemental Figure 7.jpg]

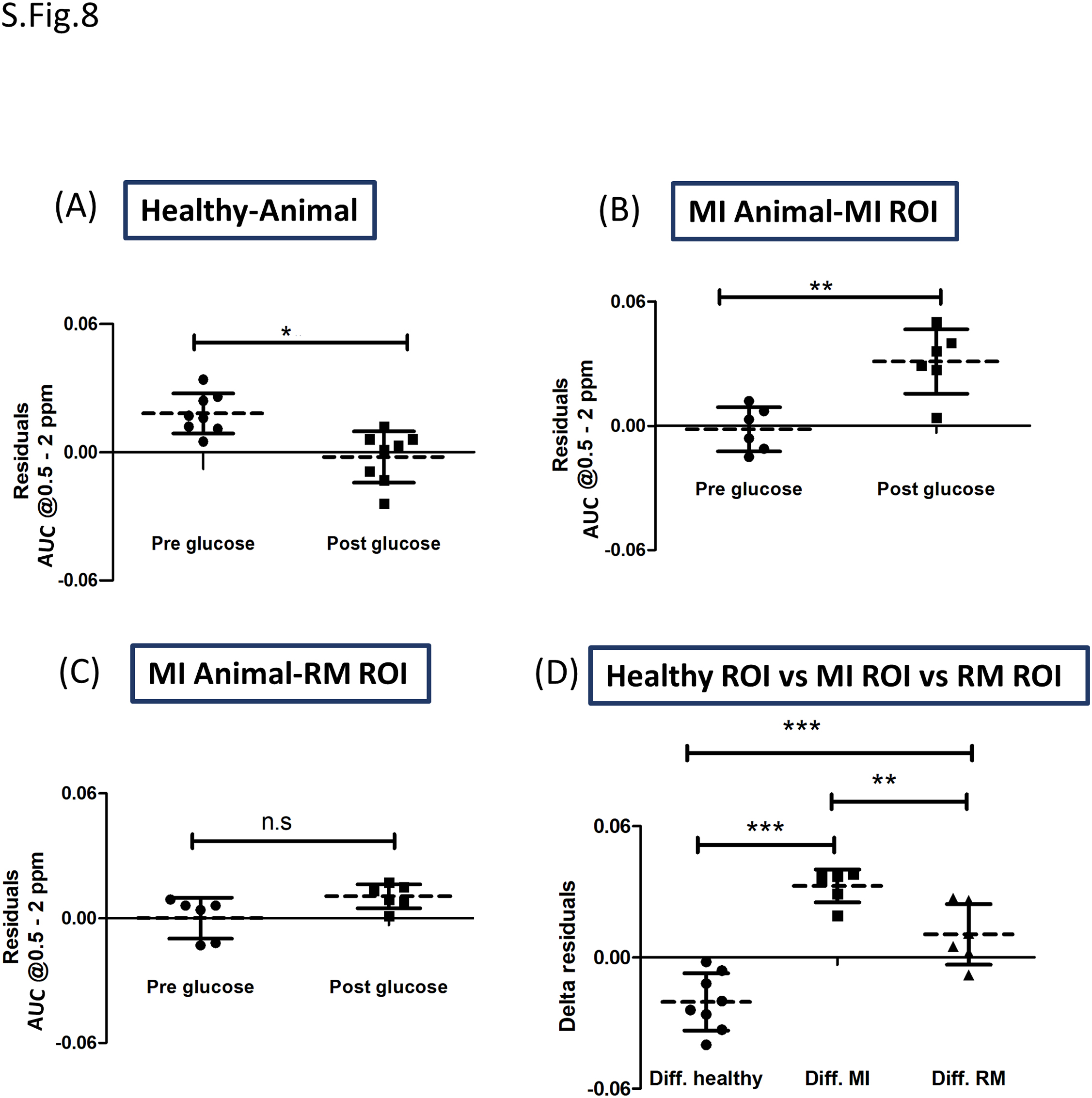

Supplement: Supplementary file 1 — Supplementary material [file mmc1.zip › Supplemental Figure 8.jpg]

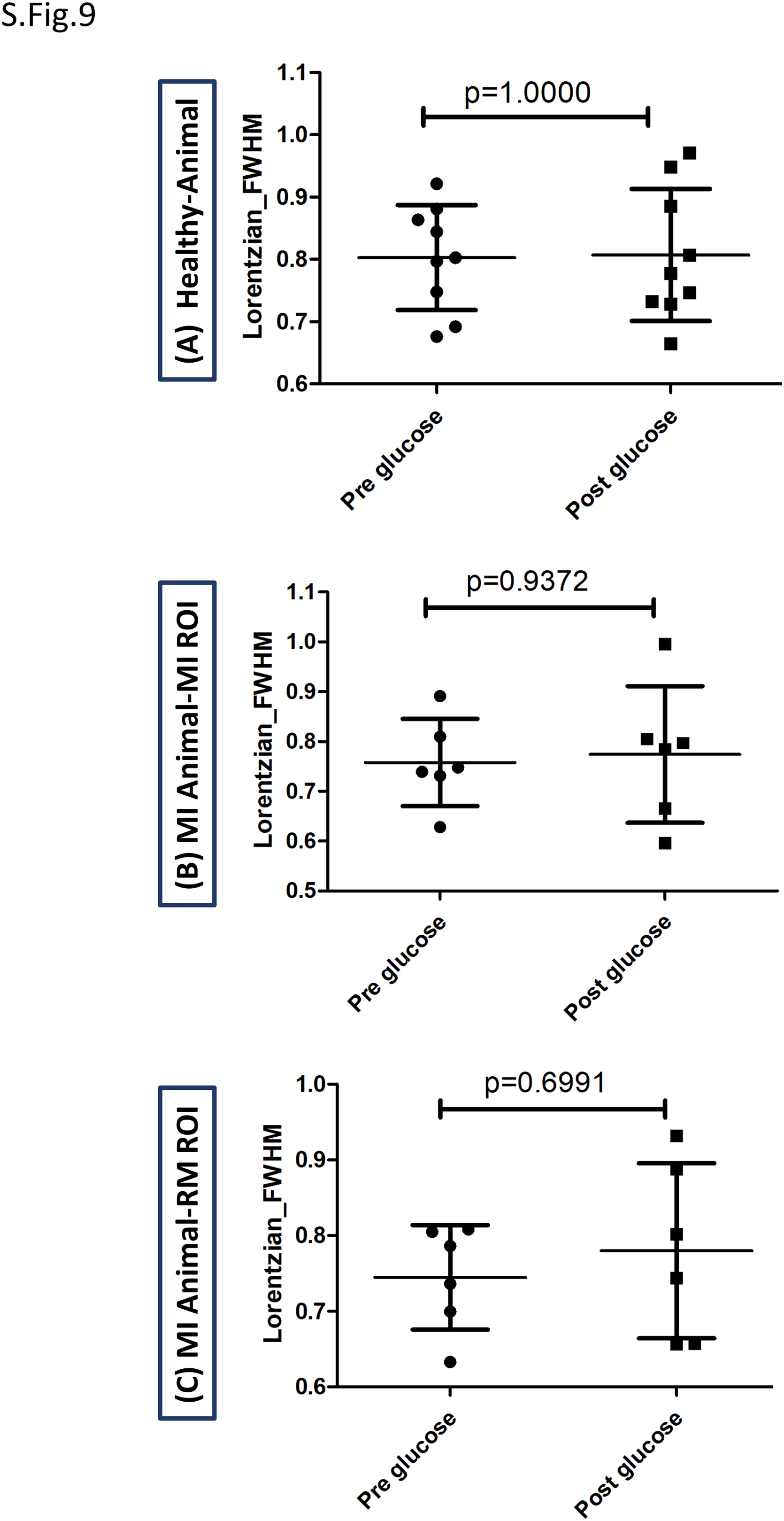

Supplement: Supplementary file 1 — Supplementary material [file mmc1.zip › Supplemental Figure 9.jpg]

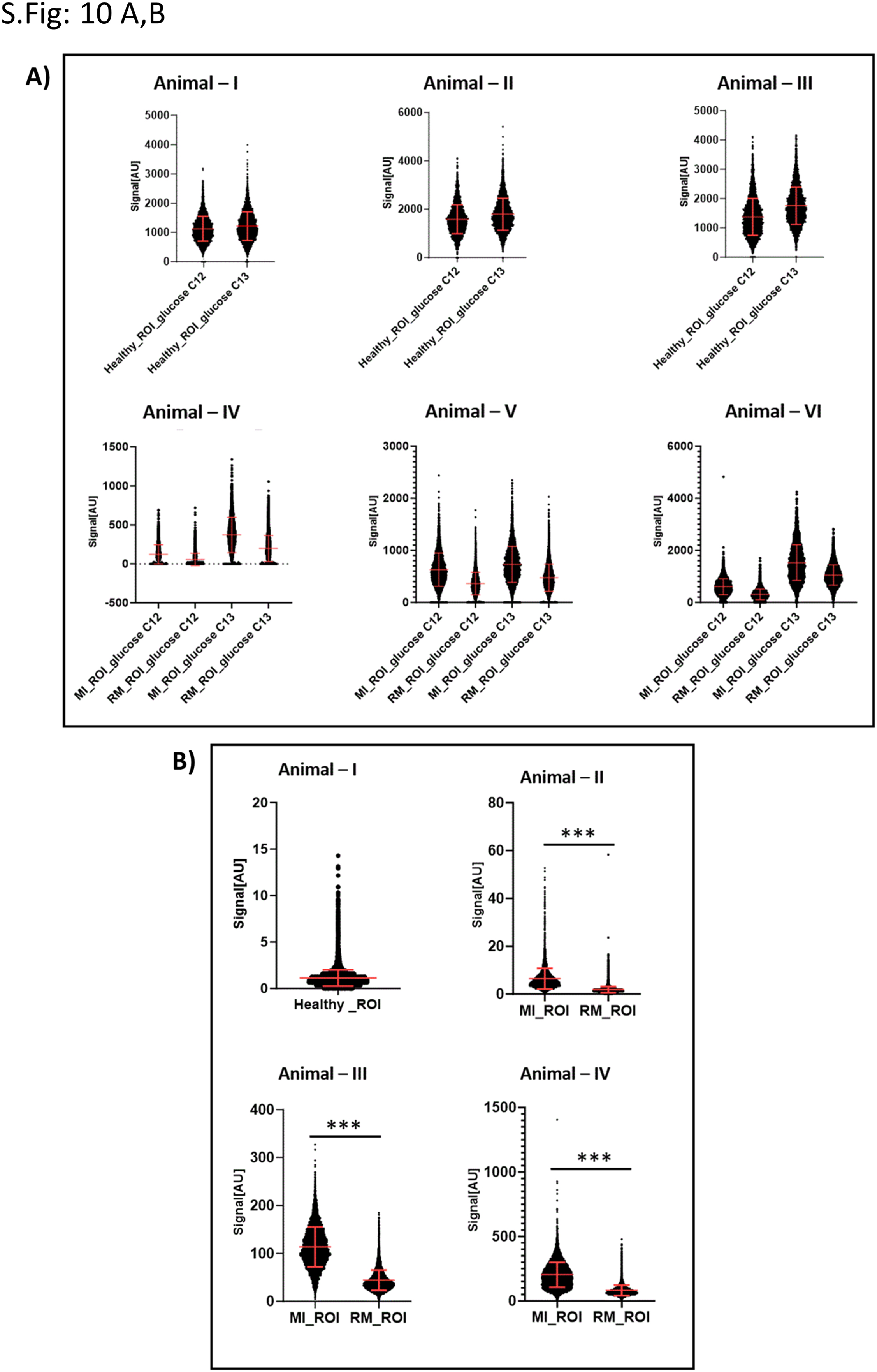

Supplement: Supplementary file 1 — Supplementary material [file mmc1.zip › Supplemental Figure 10.jpg]

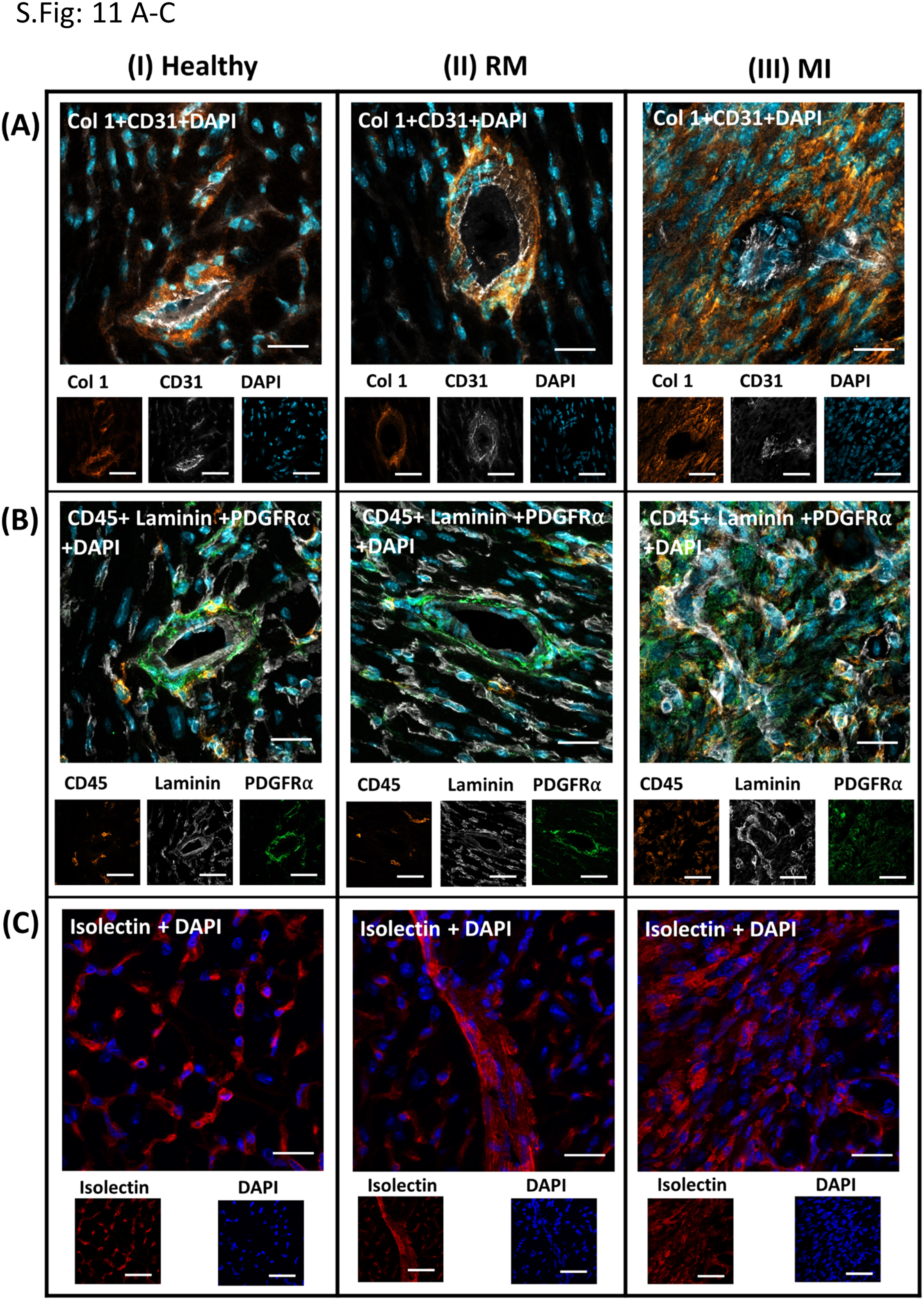

Supplement: Supplementary file 1 — Supplementary material [file mmc1.zip › Supplemental Figure 11 A-C.jpg]

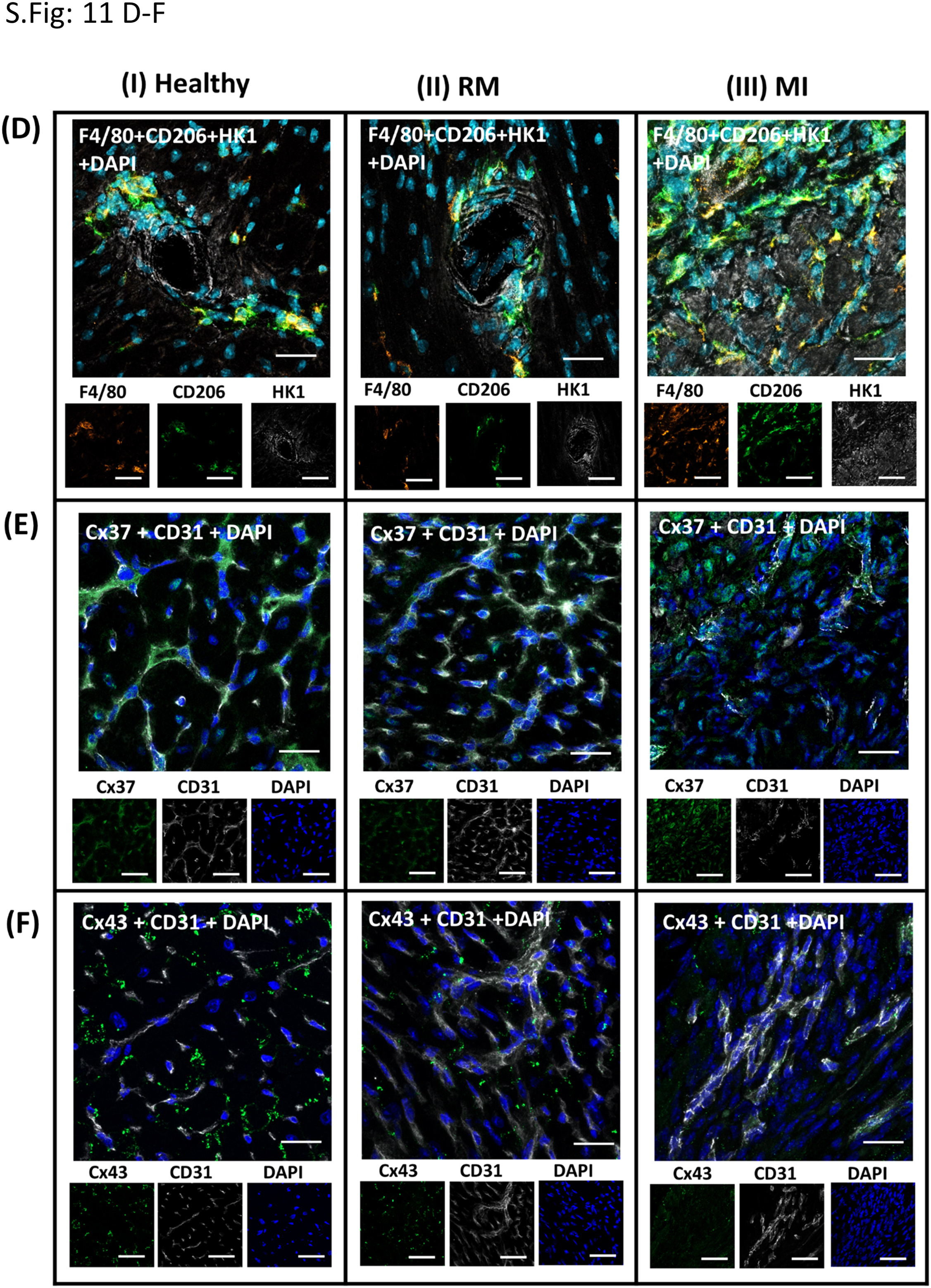

Supplement: Supplementary file 1 — Supplementary material [file mmc1.zip › Supplemental Figure 11 D-F.jpg]

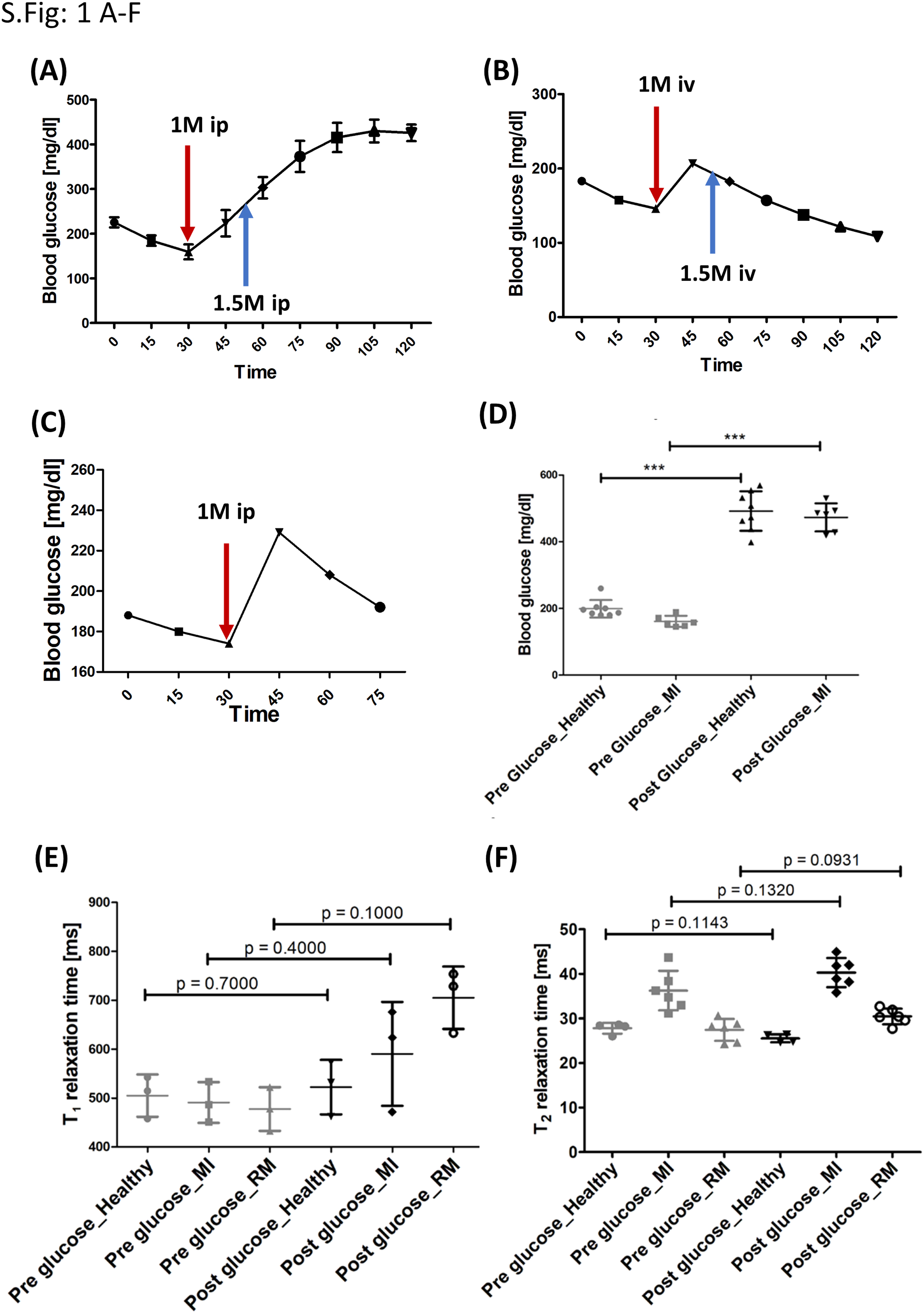

Supplement: Supplementary file 1 — Supplementary material [file mmc1.zip › Supplemental Figure 1.jpg]

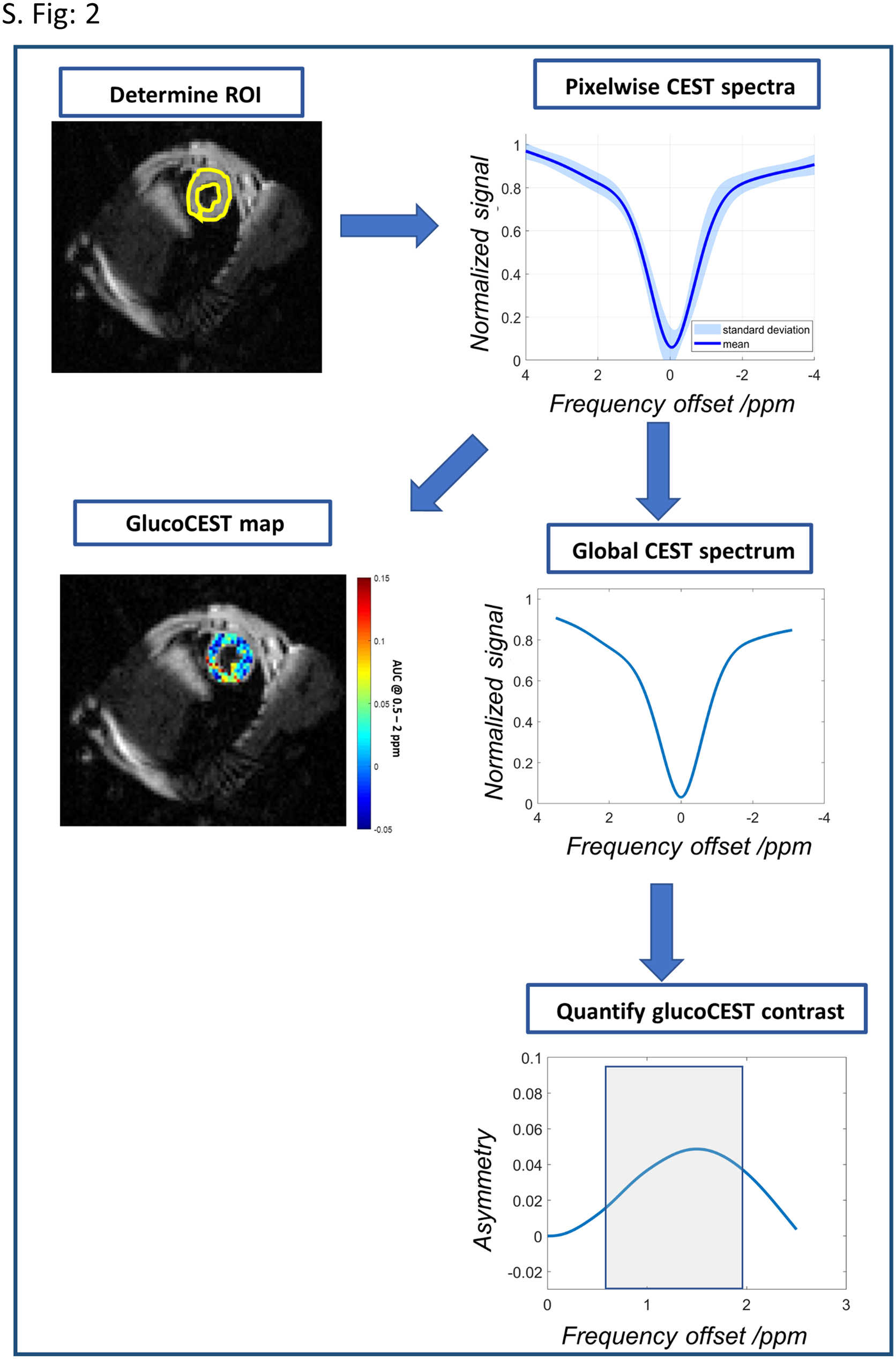

Supplement: Supplementary file 1 — Supplementary material [file mmc1.zip › Supplemental Figure 2 .jpg]

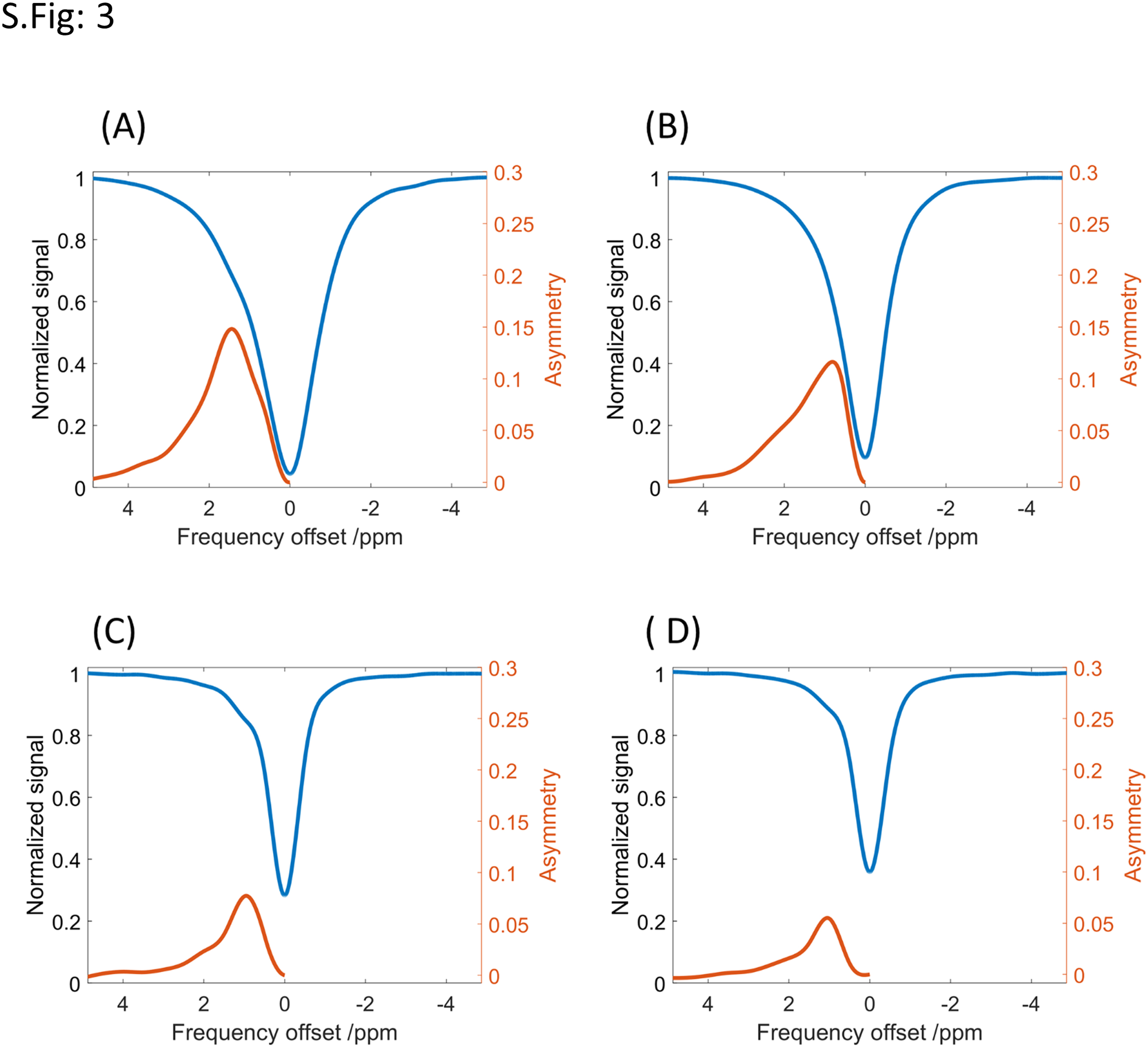

Supplement: Supplementary file 1 — Supplementary material [file mmc1.zip › Supplemental Figure 3 .jpg]
